# Thermal Stability of Au Rhombic Dodecahedral Nanocrystals Can Be Greatly Enhanced by Coating Their Surface with an Ultrathin Shell of Pt

**DOI:** 10.1021/acs.nanolett.3c02680

**Published:** 2024-01-04

**Authors:** Veronica D. Pawlik, Xiaohuan Zhao, Marc Figueras-Valls, Trenton J. Wolter, Zachary D. Hood, Yong Ding, Jingyue Liu, Miaofang Chi, Manos Mavrikakis, Younan Xia

**Affiliations:** † School of Chemistry and Biochemistry, Georgia Institute of Technology, Atlanta, Georgia 30332, United States; ‡ The Wallace H. Coulter Department of Biomedical Engineering, Georgia Institute of Technology and Emory University, Atlanta, Georgia 30332, United States; § Department of Chemical & Biological Engineering, University of Wisconsin—Madison, Madison, Wisconsin 53706, United States; ∥ School of Material Science and Engineering, Georgia Institute of Technology, Atlanta, Georgia 30332, United States; ⊥ Department of Physics, Arizona State University, Tempe, Arizona 85287, United States; # Materials Science and Technology Division, Oak Ridge National Laboratory, Oak Ridge, Tennessee 37831, United States

**Keywords:** gold, nanocrystals, platinum, plasmonics, core−shell, thermal stability

## Abstract

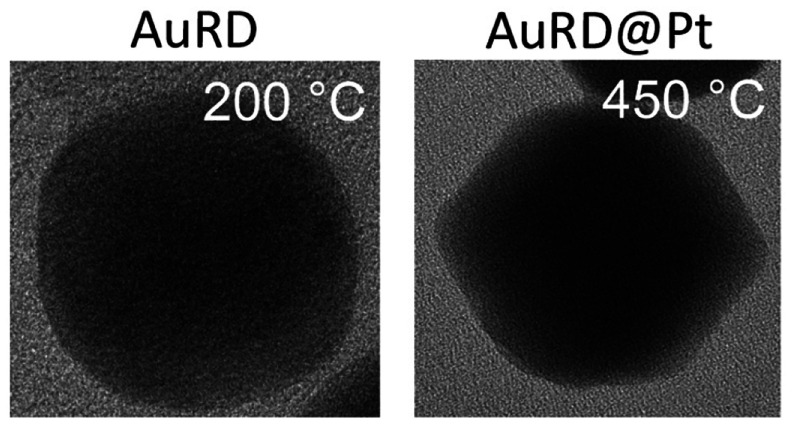

Rhombic dodecahedral nanocrystals have been considered particularly difficult to synthesize because they are enclosed by {110}, a low-index facet with the greatest surface energy. Recently, we demonstrated the use of seed-mediated growth for the facile and robust synthesis of Au rhombic dodecahedral nanocrystals (AuRD). While the unique shape and surface structure of AuRD are desirable for potential applications in plasmonics and catalysis, respectively, their high surface energy makes them highly susceptible to thermal degradation. Here we demonstrate that it is feasible to greatly improve the thermal stability with some sacrifice to the plasmonic properties of the original AuRD by coating their surface with an ultrathin shell made of Pt. Our *in situ* electron microscopy analysis indicates that the ultrathin Pt coating can increase the thermal stability from 60 up to 450 °C, a trend that is also supported by the results from a computational study.

Gold is an incredibly popular material for the colloidal synthesis of nanocrystals. Notable applications include resistance to oxidation, localized surface plasmon resonance (LSPR),^1^ biocompatibility,^2^ catalysis,^3^ self-assembly,^4^ and electrochemistry, among many others.^5^ As such, Au nanocrystals with a broad range of shapes, sizes, and internal structures have been synthesized and explored for various applications.^6−8^ Among the parameters, shape (and therefore the type of facet) is often considered the most versatile handle because of its significant impacts on both the optical and catalytic properties of the nanocrystals.^9^ For a face-centered-cubic (fcc) metal such as Au, the relative surface energies of the three low-index facets increase in the order of {111} < {100} < {110} as a consequence of the increasing degree of uncoordination for the surface atoms.^6^ As a result, the vast majority of the shapes reported for Au nanocrystals are enclosed by either {111} or {100} facets.^6^ Only within the past decade has the colloidal synthesis of Au rhombic dodecahedral nanocrystals (AuRD) solely enclosed by {110} facets been reported.^10,11^ A more thorough investigation of {110} facet formation lagged until 2021.^12^

The close-knit relationship between form and function makes it necessary to preserve the shape of nanocrystals exposed to harsh conditions such as temperature elevation.^13,14^ Particularly, Au nanocrystals are known to be susceptible to degradation at elevated temperatures because of a comparatively low melting point compounded by small particle size and high mobility of atoms.^15−17^ In addressing this issue, a number of strategies have been explored to preserve the shape of Au nanocrystals and, thereby, their catalytic and plasmonic properties. These strategies can be broadly divided into three approaches: confinement, metal–support interactions, and surface coating.^13^ Examples of confinement typically involve impregnating a prefabricated mesoporous material such as porous carbon, silica, or zeolites with the nanocrystals.^18^ Metal–support interactions can take the form of crystal defects on a TiO_2_ support, which reduce the contact angle between Au nanocrystals and the substrate.^19^ Surface coating has been widely explored to preserve the individual characteristics of nanocrystals. Presynthesized nanocrystals can be dispersed on a substrate before a thermally resistant overlayer is deposited to encapsulate the particles, forcing them to retain their original shape and position.^20^ Alternatively, core–shell nanocrystals can be formed to similarly encapsulate Au nanocrystals and protect them from degradation.^13^ However, in each of these approaches, the main goal was the preservation of size through separation or utilizing a more thermally stable material to prevent sintering and coalescence. Little regard was given to the maintenance of a specific type of facet or surface structure despite its important roles in plasmonic and catalytic applications.^21,22^

It has been difficult to precisely control the thickness of thermally stable metal oxide shells on metal nanocrystals.^13^ In contrast, a metallic shell can be readily deposited on metal nanocrystals with a thickness controllable down to one monolayer (ML). Significantly, Pt, a noble metal with a notably higher melting point than Au, has demonstrated the ability to stabilize certain types of Au surfaces beyond what would normally be expected.^23^ Herein, we report the facile synthesis of a Pt-coated AuRD using a seed-mediated route. The ultrathin Pt coating greatly improved the thermal stability of Au{110} facets from quickly disappearing at 100 °C to persisting at 450 °C even after prolonged heating. These results were further corroborated by a computational study.

The synthesis of the Pt-coated AuRD involved two steps. In the first step, AuRD were prepared using a previously reported protocol.^12^ After synthesis, the AuRD were collected by centrifugation, washed with water, and then redispersed in a small amount of *N*,*N*-dimethylformamide (DMF) before being mixed with more DMF alongside poly(vinylpyrrolidone) (PVP) and a small amount of K_2_PtCl_4_ to generate the Pt coating. Figures S1a and S1b show transmission electron microscopy (TEM) images of the 18 and 32 nm AuRD, respectively, prior to Pt coating. Note that the size was defined as the edge length. Both samples showed good uniformity in terms of shape and size. Figures S1c and S1d show TEM images of the 18 and 32 nm AuRD after Pt coating. Both images indicated no change in the uniformity of the particles alongside a negligible increase in edge length. Figure S1e provides models of RD oriented in various directions to give a better understanding of the TEM images. The optical properties of the RD were inspected by using UV–vis (Figure S2). The spectra were recorded from an ethanol suspension of the 18 nm AuRD before and after coating with Pt. As reported in the literature, AuRD exhibit three distinct LSPR peaks: one located in the mid-300 nm range, the second in the mid-500 nm range, and the last above 600 nm.^10,24^ The exact peak positions change with particle size, but they can be assigned to the octupole, quadrupole, and dipole modes, respectively. The peak at 540 nm was slightly blue-shifted from the peak we previously reported at 547 nm, which can be ascribed to the decrease in particle size.^13,26^ This peak at 540 nm did not show any shift after Pt coating, albeit its intensity decreased as a result of plasmon damping associated with the Pt coating. The shoulder peak located at 638 nm increased in intensity and red-shifted to 701 nm after the Pt coating. In the case of thin Pt shells on plasmonic cores, red-shifts are common and indeed serve as another indication of the presence of an ultrathin Pt shell in our system.^25,26^ Although the damping may hinder some potential plasmonic applications, the activity remains reasonably high.

Because the atomic numbers of Au and Pt are so close, it is difficult to distinguish the Pt layer through a change in contrast on the TEM images alone. Instead, high angle annular dark-field scanning TEM (HAADF-STEM) images were taken from the 18 nm Pt-coated AuRD (Figure 1a). The corresponding energy-dispersive X-ray spectroscopy (EDX) elemental mapping in Figure 1b was recorded from the area outlined by a green box in Figure 1a. The mapping was restricted to this area to maximize the signal obtained from Pt. From the model shown as an inset in Figure 1a, it is apparent that the only facets parallel to the electron beam are those on the left and right of the nanocrystal’s hexagonal projection. Because the Pt coating is only 1 ML in thickness, it is very difficult to collect enough Pt signals head-on. However, if the coating is observed along the two facets, the “thickness” as observed from the perspective of the electron beam greatly increases. The Pt and Au maps in Figure 1b indicate a strict elemental segregation and exceedingly thin Pt shell. Figure 1c shows an EDX line scan of the elemental distribution taken along the path marked by the red line in Figure 1a. The complementary profiles of Pt and Au further confirm a core–shell structure. Figures 1d and 1e show a high-resolution TEM (HRTEM) image of another 18 nm Pt-coated AuRD and the corresponding selected area electron diffraction (SAED) pattern before heating, respectively. The change in contrast across the nanocrystal in Figure 1d forms a darker diamond shape at the center of the RD. This shape is consistent with the model shown in the inset of Figure 1a, viewed along the [011] zone axis. Further, the lattice fringe makes it possible to identify the left and right facets as {110}. The diffraction spots in Figure 1e confirm this facet assignment.

**Figure 1:**
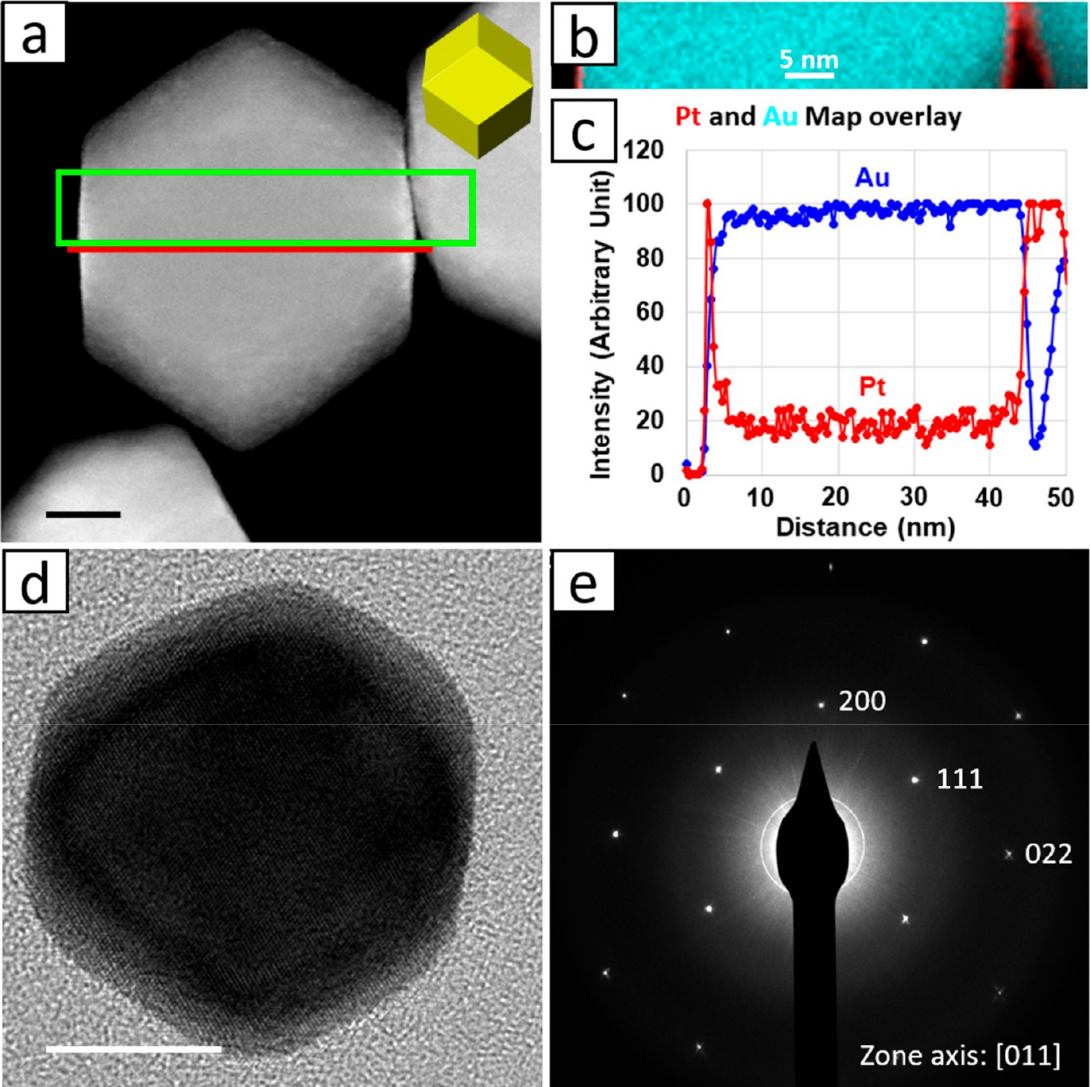
(a) HAADF-STEM image with an area boxed in green for EDX mapping and a red line to mark the path for the EDX line scan, with the inset showing a model RD in the same orientation as the imaged nanocrystal. (b) EDX elemental mapping of Au (teal) and Pt (red). (c) EDX line scan. (d) HRTEM image of an 18 nm Pt-coated AuRD. (e) Corresponding SAED pattern with diffraction spots labeled. Scale bars: 10 nm.

However, it should be noted that both atomic resolution TEM and HAADF-STEM imaging methods are not completely reliable in differentiating Au from Pt due to their closeness in atomic number. The presence of strain-field-induced scattering and edge scattering of electrons complicates the interpretation of the atomic resolution profile images. For the present system, EDX mapping also has issues because of the closeness of the X-ray peaks of Au and Pt, leading to uncertainties in the accurate measurement of the ultrathin Pt layer. For plan-view EDX mapping, the weak X-ray signal from the ultrathin Pt layer and the strong X-ray signal from the Au substrate introduce large errors in quantitatively evaluating the exact thickness of the ultrathin Pt layer. Taken together, we do not have a reliable method based on electron microscopy for measuring the exact thickness of the ultrathin Pt shell on Au. Alternatively, to further confirm the presence of Pt on the AuRD surface, we analyzed the Pt-coated particles by using X-ray photoelectron spectroscopy (XPS) and inductively coupled plasma mass spectroscopy (ICP-MS). The XPS data in Figure S3 confirm the presence of Pt in the sample. The ICP-MS data (see the Supporting Information for a detailed calculation) indicate that the average thickness of the Pt shell was approximately 1 ML.

To demonstrate the positive impact of the added ultrathin Pt shell, we first characterized the thermal stability of pristine AuRD. Figure S4 shows TEM images of the 18 nm AuRD after being heated in DMF at temperatures ranging from 60–120 °C and for 3 h at each temperature. After heating at 60 °C, a temperature 30 °C below what was used for the synthesis of AuRD, minor corner rounding was observed, but the overall shape and facets were largely intact (Figure S4a). Increasing the temperature to 80, 100, and 120 °C resulted in an increase in corner roundness until the shape evolved into a flattened spherical shape (Figure S4b–d). Figure S5 shows the results from the same experiment performed on the 32 nm AuRD. The larger size offered a greater resistance to degradation (corner rounding only became noticeable at 120 °C (Figure S5d)); however, the overall trend repeated. This increased stability is attributed to the larger number of atoms necessary to diffuse to achieve the same degree of rounding in a larger nanocrystal.

As described in the Experimental Section, Pt coating was conducted at 130 °C. Consistent with the trend observed in Figure S4, Figure 2a indicates that heating the pristine 18 nm AuRD at such a high temperature for 3 h resulted in a complete loss of the original shape and facet definition. After Pt coating, the products were again subjected to prolonged heating in solution, this time in the range 130–180 °C and for 3 h at each temperature. Figure 2b–d shows TEM images of the Pt-coated AuRD after heating. It is clear, particularly from the insets, that the Pt-coated AuRD retained their original shape and facets very well even when heated at 180 °C for 3 h. Figure S6 shows the corresponding results for the 32 nm AuRD. Once again, much definition was lost when the pristine nanocrystal was heated to 130 °C (Figure S6a). Likewise, coating the AuRD with an ultrathin Pt layer resulted in substantial thermal protection (Figure S6b–d). For this reason we believe that the Pt deposition occurred rapidly, as the coated nanocrystals do not exhibit degradation, which should be apparent at the coating temperature.

**Figure 2:**
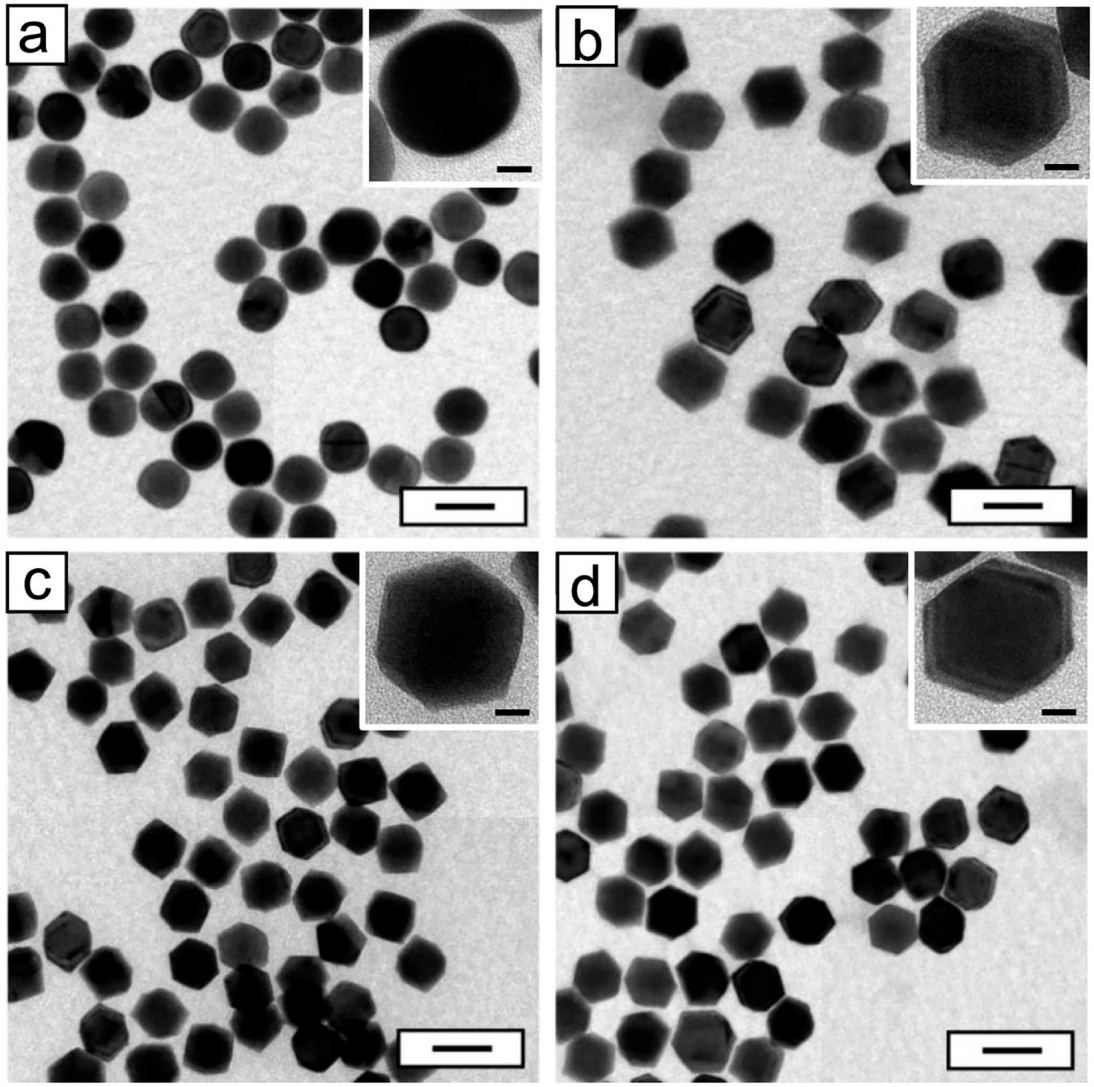
(a) TEM image of the 18 nm AuRD after being heated in DMF at 130 °C for 3 h. (b–d) TEM images of the 18 nm Pt-coated AuRD after being heated in DMF at (b) 130, (c) 150, and (d) 180 °C and for 3 h at each temperature. Scale bars: 50 nm. Scale bars in the insets: 10 nm.

We also analyzed the shape degradation of the AuRD by heating the nanocrystals *in situ* under a TEM. Figure 3 details the shape evolution of a single 18 nm AuRD as it was heated from 25 to 200 °C in approximately 20 °C intervals and for 20 min at each temperature. As shown in Figure 3a,b, the AuRD shows flat facets and no corner rounding. At 60 °C (Figure 3c), the first signs of delicate corner rounding appeared, most notably observed on the rightmost corner. However, the facets remained flat, retaining their {110} character. At 80 °C, corner rounding increased, becoming more obvious, and the first signs of facet rounding also appeared (Figure 3d). At this point, the RD shape was still distinguishable but severely degraded. When the temperature was increased to 100 °C, both corner and facet rounding increased, and the degree of facet rounding indicates a loss of distinct {110} facets, and the original shape is considered lost (Figure 3e). Further heating to 200 °C continued shape degradation until the nanocrystal took on a spherical shape (Figure 3f–i). The same *in situ* TEM heating was also performed on a 32 nm AuRD, and the results are shown in Figure S7. As observed earlier, the increased size of the AuRD gave the nanocrystal improved thermal stability; however, the overall trend in shape degradation remained the same. Up to 80 °C, the RD shape was maintained relatively well with some minimal corner rounding (Figure S7a–c). At 100 °C, some facet rounding started to become apparent (Figure S7d). Both corner and facet roundings increased until the shape is considered lost at 140 °C (Figure S7f). After this point, the distinct hexagonal projection quickly disappeared, and the nanocrystal became spherical at 200 °C (Figure S7i).

**Figure 3:**
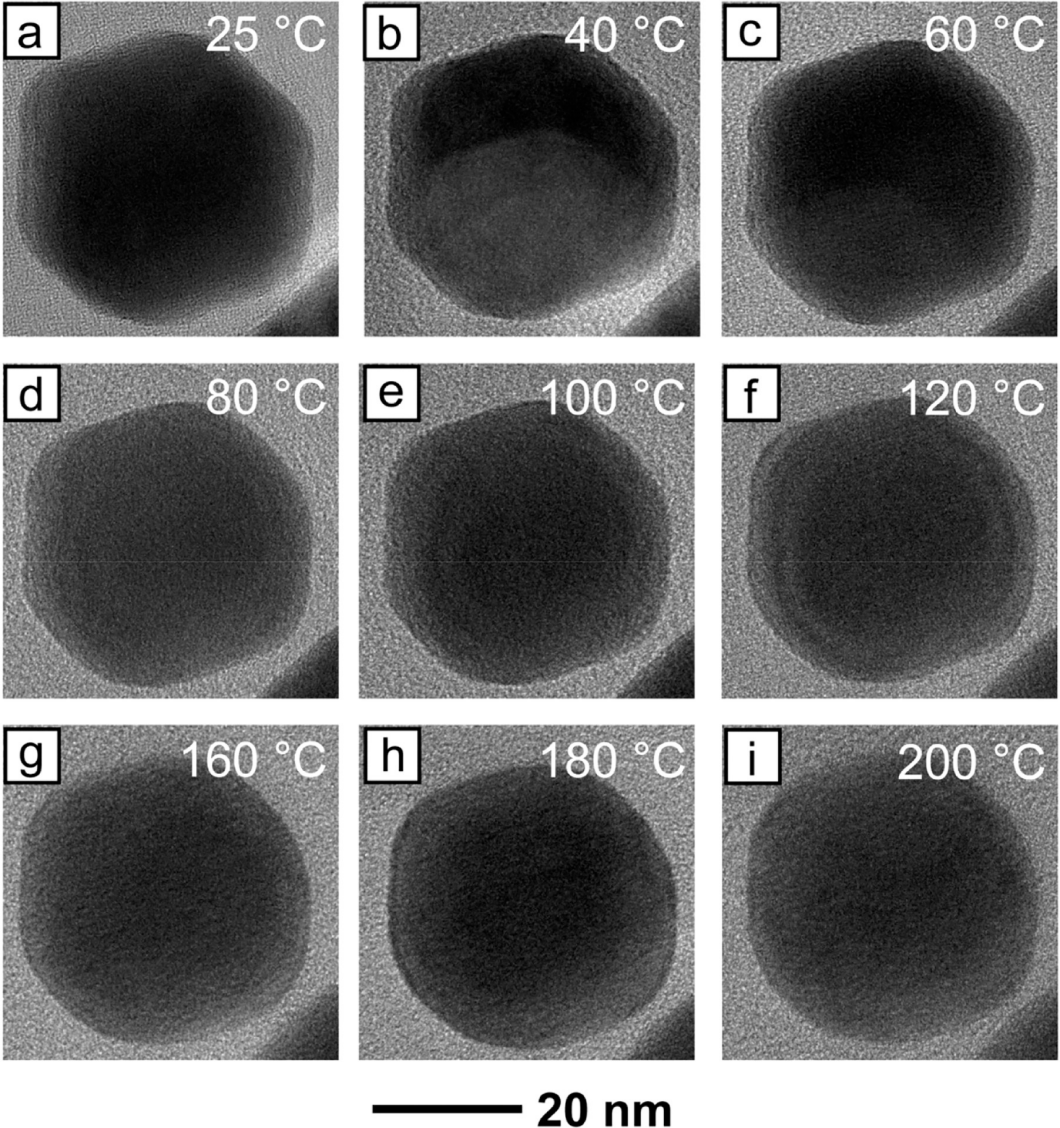
*In situ* TEM images recorded from the 18 nm AuRD upon heating consecutively at different temperatures for 20 min: (a) 25, (b) 40, (c) 60, (d) 80, (e) 100, (f) 120, (g) 160, (h) 180, and (i) 200 °C. The original shape was lost at 100 °C, and the nanocrystal evolved into a nanosphere at the end.

When the *in situ* TEM heating was repeated on the Pt-coated AuRD, a significant increase in the thermal stability was observed. Whereas the 18 nm AuRD began to show signs of corner rounding at a temperature as low as 60 °C, no corner rounding or loss of facet definition was apparent in the 18 nm Pt-coated AuRD until they were heated to 400 °C (Figure 4). Even at this temperature, indications of shape degradation were still mild, and distinct corners and flat facets were easily observable. When temperature was further increased to 450 °C, a bit more corner rounding became apparent, but {110} facets were still intact, and the hexagonal projection remained clear (Figure 4i). Interestingly, the 32 nm Pt-coated AuRD did not exhibit a notable increase in thermal stability, as was previously observed in the heating experiment involving pristine AuRD (Figure S8). Similar to the 18 nm counterpart, the 32 nm Pt-coated AuRD began to lose its shape at 400 °C (Figure S8h) with some more degradation apparent when the temperature was increased to 450 °C (Figure S8i). The final nanocrystal was still identifiable as an RD. This is likely the product of two factors. First, the initial corners on the chosen 32 nm Pt-coated AuRD were not as sharp as those of the 18 nm counterpart (Figure S8a versus Figure 4a). This makes any impact of corner rounding on shape degradation more obvious. Second, in both heating ramps, the temperature jumped from 350 to 400 °C. Thus, it is difficult to pinpoint at what temperature exactly each nanocrystal began to exhibit corner rounding.

**Figure 4:**
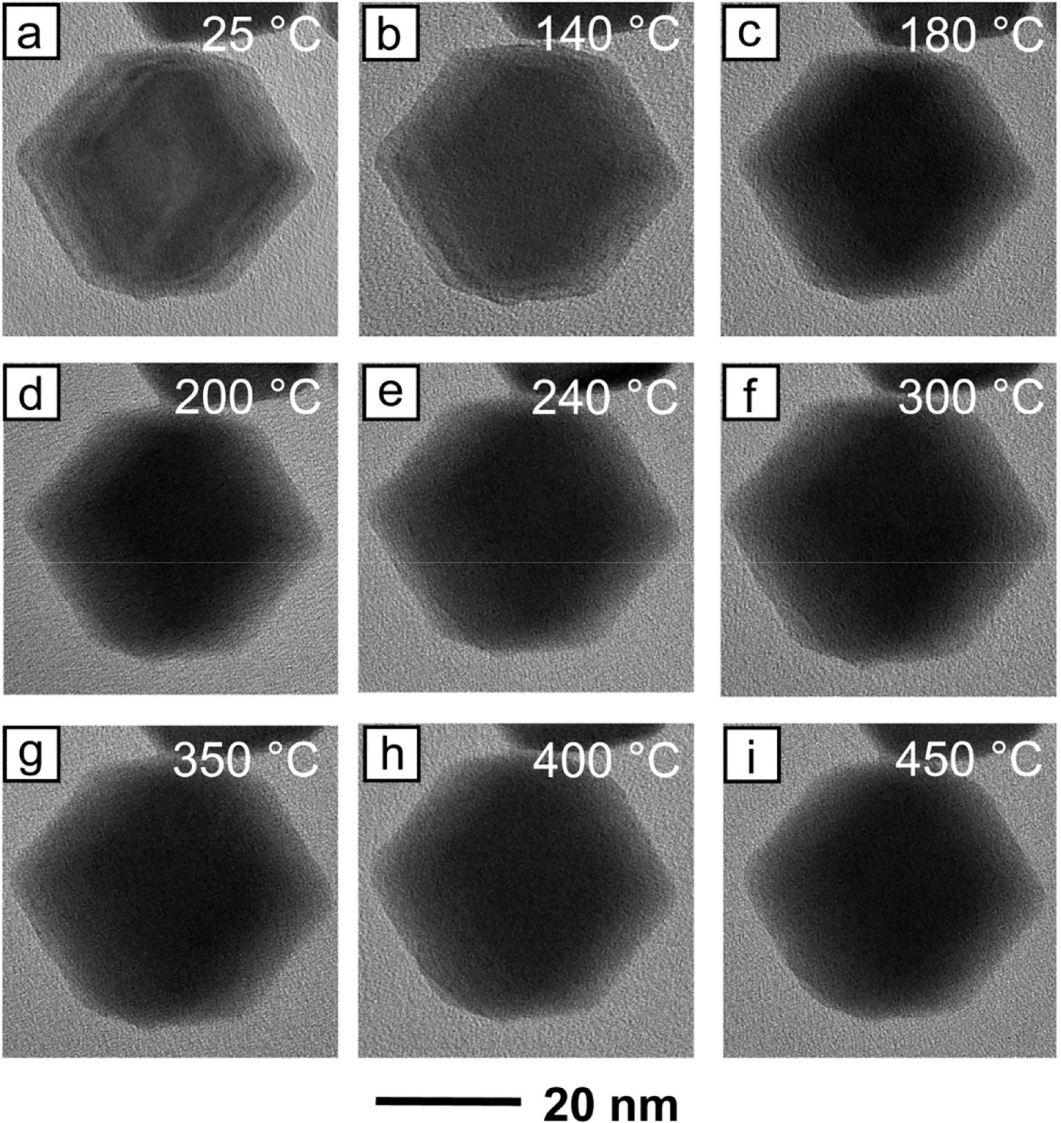
*In situ* TEM images recorded from the 18 nm Pt-coated AuRD upon heating consecutively at different temperatures for 20 min: (a) 25, (b) 140, (c) 180, (d) 200, (e) 240, (f) 300, (g) 350, (h) 400, and (i) 450 °C. It is worth noting that the original shape was not lost until 400 °C.

We conducted another TEM study to better characterize the Pt-coated AuRD facets after heating. Figures S9a–e show TEM images of a 32 nm Pt-coated AuRD heated from room temperature to 450 °C. The continued presence of a darker diamond contrast at the center of the RD throughout heating is a good indication of the preservation of the shape and facets. Figure S9f shows an HRTEM image of the same nanocrystal taken upon cooling to room temperature. The FFT pattern indicates that the image was taken along the [011] zone axis. This orientation means that the two flatter faces of the RD (top right and bottom left) are {110} facets. Figures S9g and S9h show a closer look at the {110} and {100} facets, respectively, as marked by the red (Figure S9g) and green (Figure S9h) outlines in Figure S9f. Despite being heated to 450 °C, the 32 nm Pt-coated AuRD was clearly able to retain flat {110} facets. This study confirms that the previously observed corner rounding corresponds to the appearance of {100} facets.

To better understand the disparity in thermal stability between monometallic Au and Pt-coated AuRD nanocrystals, a computational study was performed. The thermal stability of both AuRD and Pt-coated AuRD was assessed by calculating the ejection energy, which is the lowest energy required to eject a surface Au or Pt atom, respectively. In this context, the ejection energy serves as a descriptor of the stability toward reconstruction of the nanocrystal. To ensure the relevance of the calculated ejection energies, several models of the (110) surface were considered, which is the primary facet displayed on the Au@Pt nanocrystals. These models account for the presence of 1–4 ML of Pt atoms supported on an Au(110) surface (Figure S10) and the existence of moiré patterns, which represent nonstoichiometric Pt overlayers whereby the numbers of overlayer (Pt) and underlayer (Au) atoms are not equal (Figure S11 and Table S1). First, the relative stability of each overlayer pattern was evaluated by calculating its surface energy (Γ). Subsequently, the ejection energy was computed for the Pt overlayer exhibiting the smallest Γ (Figures 5 and S12). All necessary calculations were performed with density functional theory (DFT) and machine learning interatomic potentials (IPs). Details of the computational methods and relevant parameters are provided in the Supporting Information.

**Figure 5:**
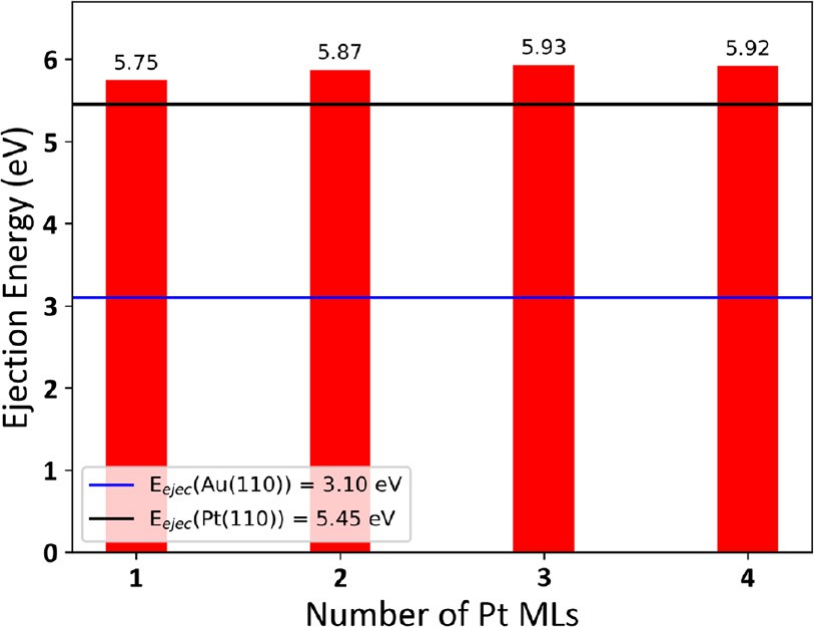
DFT-calculated ejection energies for Pt atoms in the Pt overlayer (*P* = 1) supported on Au(110). For comparison, blue and black horizontal lines indicate the DFT-calculated ejection energies of Au and Pt atoms from a monometallic (110) surface, respectively.

The ejection energy was calculated for the most stable overlayer pattern with DFT and IP. Figure 5 shows that, per DFT, the ejection of a Pt atom from a stoichiometric Au@Pt surface is more difficult than that from a Pt(110) slab model surface and at least 2.65 eV more difficult than the ejection of an Au atom from an Au(110) slab model surface, which is in agreement with our experimental observation. Moreover, as the number of Pt ML on the Au surface increases, the Pt atom ejection energy keeps increasing and stabilizes when the number of Pt ML becomes 3 or 4. Importantly, at room temperature, every 0.06 eV in energy translates to an order of magnitude in dissolution rates. Notably, a similar trend for Au@Pt overlayers was observed in the (111) geometry.^23^ Specifically, Lopes et al. observed that for nonstoichiometric overlayers of Pt on Au(111), the ejection energy of the Pt atom for cases with 1 and 4 ML of Pt was higher than for ejection from monometallic Pt(111). We attribute this increase to the bonding between Au and Pt at the interface in the Au@Pt system and the stabilization imparted by the strain fields induced by overlaying Pt on Au.

In summary, we have developed a simple approach to drastically improve the thermal stability of AuRD by coating their surface with an ultrathin shell of Pt. Specifically, the Pt-coated AuRD was able to retain the characteristic LSPR features of the AuRD, indicating a relatively narrow size distribution and good preservation of shape after coating. This was further confirmed visually by TEM images. While the pristine AuRD began to degrade slowly at temperatures as low as 60 °C, the Pt-coated AuRD were able to preserve the RD shape up to 450 °C. The drastic improvement in thermal stability was also accounted for through a computational study. Consequent preservation of the LSPR properties makes these nanocrystals ideal candidates for high-temperature applications that require stable optical conditions. The coating method is robust and was successfully applied to both 18 and 32 nm AuRD. This study demonstrates a new avenue for stabilizing the less stable shapes of metal nanocrystals for use in harsher and more demanding applications. In this way, the desirable properties of unstable nanocrystals can be more widely adopted.

## Supporting Information Available

Experimental section, computational section, additional TEM characterizations of AuRD before and after coating with Pt, UV–vis spectra of AuRD before and after coating with Pt, TEM, HRTEM images of the 32 nm AuRD (with and without Pt coating) for the analysis of thermal stability, and data from computational modeling (PDF)
