# Supplementary material for: Thermal Stability of Au Rhombic Dodecahedral Nanocrystals Can Be Greatly Enhanced by Coating Their Surface with an Ultrathin Shell of Pt
Source: Nano Lett. 2024 Jan 4;24(2):549–56. doi: 10.1021/acs.nanolett.3c02680 (PMC10797619; doi:10.1021/acs.nanolett.3c02680)
Supplement: Supplementary file 1 — nl3c02680_si_001.pdf [file nl3c02680_si_001.pdf]

Supporting Information

The Thermal Stability of Au Rhombic Dodecahedral Nanocrystals Can Be Greatly Enhanced by Coating Their Surface with an Ultrathin Shell of Pt

Veronica D. Pawlik[a,†], Xiaohuan Zhao[b,†], Marc Figueras-Valls[c,†], Trenton J. Wolter[c], Zachary D. Hood[a], Yong Ding[d], Jingyue Liu[e], Miaofang Chi[f], Manos Mavrikakis[c,*], and Younan Xia[a,b,*]

[a]*School of Chemistry and Biochemistry, Georgia Institute of Technology, Atlanta, Georgia 30332, United States*

[b]*The Wallace H. Coulter Department of Biomedical Engineering, Georgia Institute of Technology and Emory University, Atlanta, Georgia 30332, United States*

[c]*Department of Chemical & Biological Engineering, University of Wisconsin –Madison, Madison, WI 53706, United States*

[d]*School of Material Science and Engineering, Georgia Institute of Technology, Atlanta, Georgia 30332, United States*

[e]*Department of Physics, Arizona State University, Tempe, Arizona 85287, United States*

[f]*Materials Science and Technology Division, Oak Ridge National Laboratory, Oak Ridge, Tennessee 37831, United States*

*Email: emavrikakis@wisc.edu, younan.xia@bme.gatech.edu

Experimental Section

Materials. Tetrachloroauric(III) acid ($HAuCl_4 \cdot 3H_2O$, ≥99.0%), N,N-dimethylformamide (DMF, ≥ 99%), sodium borohydride ($NaBH_4$, ≥99%), poly(vinylpyrrolidone) (PVP, MW≈55 000), L-ascorbic acid (AA, >99%), hexadecyltrimethylammonium chloride (CTAC, 25% wt./vol. aqueous solution), hexadecyltrimethylammonium bromide (CTAB, ≥99%), and potassium tetrachloroplatinate (K_2PtCl_4, ≥99.99%) were all obtained from Sigma-Aldrich (St. Louis, MO) and used as received. Deionized water with a resistivity of 18.2 MΩ·cm at room temperature was used throughout the experiments.

Characterizations. TEM images were obtained using a JEOL-1400 or Hitachi HT7700 electron microscope operated at 120 kV. Samples were prepared by dropping aqueous suspensions of the particles on carbon-coated copper grids, followed by drying under ambient conditions. HAADF-STEM images and energy-dispersive X-ray (EDX) spectroscopy data were acquired at Arizona State University using a JEOL JEM 2200FS scanning TEM (STEM) microscope equipped with a CEOS probe corrector (Heidelberg, Germany) to provide a nominal image resolution of 0.07 nm. *In situ* heating studies were done using a Protochips Aduro heating holder in a Cs-corrected FEI Titan 80/300 kV TEM/STEM microscope at Oak Ridge National Laboratories (ORNL). The images were acquired at 300 kV with a beam size of 0.07 nm. The samples were prepared by dropping the colloidal nanocrystals onto a Protochips E-chip for Aduro and allowing the specimen to dry under ambient conditions before imaging. Heat ramping was done at a rate of 1000 °C/ms to simulate instantaneous temperature rise. SAED patterns and corresponding HRTEM and STEM images were taken using a Tecnai G2 F30 S-TWIN microscope operated at 300 kV. *In situ* heating studies were also performed using a Gatan Model 652 double tilt holder equipped with a Gatan Model 901 SmartSet Hot Stage Controller. X-ray photoelectron spectroscopy (XPS) analyses were conducted using Thermo K-Alpha XPS. UV-vis spectra were recorded on a Cary 60 spectrometer (Agilent). The Au and Pt contents in a sample were determined using an inductively-coupled plasma mass spectrometer (ICP-MS, NexION 300Q, PerkinElmer). The sample was prepared by dissolving the particles in a 3:1 mixture of hydrochloric acid (HCl, 37% by vol.) and nitric acid (HNO_3, 70% by vol.).

Synthesis of Au Spherical Seeds. The protocol for the synthesis of 10-nm Au spherical seeds was

reported elsewhere.[1] Briefly, Au clusters were synthesized by mixing 0.6 mL of a 10 mM aqueous $NaBH_4$ solution with 10 mL of 0.25 mM aqueous $HAuCl_4$ and 0.1 M aqueous CTAB in a 20 mL vial. The solution was then gently shaken for 2 min before being left undisturbed at 27 °C for 3 h. Afterward, 1.5 mL of 0.1 M aqueous AA was added to a separate vial containing 4 mL of 0.25 mM aqueous $HAuCl_4$, 4 mL of 0.1 mM aqueous CTAC, and 0.1 mL of the as-prepared Au clusters. This reaction was allowed to proceed for 1 h, after which the solid products were collected by centrifugation at 14500 rpm for 30 min and washed with water. The as-obtained Au nanospheres were about 10 nm in diameter. The particles were then redispersed in water at a concentration of 10.66 mg/mL, confirmed using ICP-MS, to match the protocol reported in literature.[2]

Synthesis of 18- and 32-nm AuRD. The AuRD were synthesized as reported previously.[2] Briefly, 200 mg of PVP was dissolved in 6 mL of DMF in a 20 mL vial at room temperature before the addition of 10 μL of the 10-nm Au nanospheres alongside 25 μL of 40 mM aqueous $HAuCl_4$. The vial was then briefly shaken before being placed in an oil bath set to 90 °C under magnetic stirring. The vial was removed after 30 min to obtain 18-nm AuRD or after 60 min to obtain 32-nm AuRD. Once removed, the vial was immediately immersed in an ice-bath to quench the reaction. The AuRD were collected *via* centrifugation at 14,500 rpm for 5 min. The products were subsequently washed with water three times in the same fashion before being redispersed in water for further characterizations.

Synthesis of Pt-coated AuRD. The pre-synthesized AuRD was centrifuged at a speed of 14,500 rpm for 5 min to collect the solid products, which were then redispersed in water and centrifuged again in the same manner. The AuRD were then resuspended in DMF to a final volume of 50 μL. The concentrated AuRD were added into a 20-mL vial containing 6 mL of DMF and 60 mg of PVP. Finally, 25 μL of 40 mM K_2PtCl_4 dissolved in DMF was added before shaking the vial and placing it in an oil bath set to 130 °C. The reaction was allowed to proceed for 3 h before being quenched in an ice bath. The solid products were collected through centrifugation at 14,500 rpm for 5 min before being washed with water three times and finally redispersed in water for further characterizations.

ICP-MS Calculations

The concentrations of Au and Pt as measured by ICP-MS are 17.26 mM and 0.91 mM, respectively. In 100 μL of the sample, these correspond to 3.4x10^{-4} g of Au and 1.8x10^{-5} g of Pt.

$$Au: (0.01726\ M)(0.0001\ L) = 1.726 \cdot 10^{-6}\ mol$$

$$(1.726 \cdot 10^{-6}\ mol)(196.96\ g/mol) = 3.4 \cdot 10^{-4}\ g$$

$$Pt: (0.00091\ M)(0.0001\ L) = 9.099 \cdot 10^{-8}\ mol$$

$$(0.9099 \cdot 10^{-8}\ mol)(195.08\ g/mol) = 1.8 \cdot 10^{-5}\ g$$

These gram values are then converted to volumes through density:

$$Au: \frac{3.4 \cdot 10^{-4}\ g}{19.30\ g/cm^3} = 1.76 \cdot 10^{-5}\ cm^3 = 1.76 \cdot 10^{16}\ nm^3$$

$$Pt: \frac{1.8 \cdot 10^{-5}\ g}{21.45\ g/cm^3} = 8.27 \cdot 10^{-7}\ cm^3 = 8.27 \cdot 10^{14}\ nm^3$$

The volume of an RD is given by:

$$Volume_{RD} = \frac{16\sqrt{3}}{9} s^3$$

where s is the edge length. The average edge length derived from TEM images of the sample is 18.0 nm, thus, the volume of one AuRD is 17957.9 nm^3. To find the number of nanocrystals, the total volume of Au is divided by the individual volume of an AuRD:

$$Number\ of\ AuRD = \frac{V_{RD\ 18nm}}{V_{Au}} = 9.8 \cdot 10^{11}$$

To find the additional volume introduced by the Pt shell, the volume of Pt is divided by the number of nanocrystals:

$$Volume\ Pt\ per\ RD = \frac{V_{Pt}}{Number\ of\ AuRD} = 843.3\ nm^3$$

The new edge length can be calculated by adding the original AuRD volume to the additional volume contributed by Pt and then using the RD volume equation to solve for s:

$$New\ edge\ length = \sqrt[3]{\frac{9V_{total}}{16\sqrt{3}}} = 18.277\ nm$$

The thickness of a layer can be obtained by calculating the {220} *d* spacing for Pt:

$$\frac{1}{d^2} = \frac{h^2 + k^2 + l^2}{a^2} = \frac{2^2 + 2^2 + 0^2}{0.392^2\ nm}$$

This gives a d spacing of 0.138 nm. The difference in edge length can then be divided by the d spacing to calculate the number of atomic layers.

$$Atomic\ layers = \frac{0.277\ nm}{0.138\ nm} = 2.002$$

Finally, the number of atomic layers is divided by 2 to give the number of monolayers.

$$Monolayers = \frac{2.002}{2} = 1$$

Computational details

The (110) surface is highly corrugated, with its corrugation being especially prominent in the X-axis direction, as depicted in Figure S10a. This corrugation makes it nontrivial to explore bidirectional non-stoichiometric overlayers, as the Pt overlayer would induce highly uncoordinated atoms and defects in the X-axis direction. However, the surface height remains unperturbed in the Y direction, facilitating the accommodation of a non-stoichiometric overlayer whereby the number of atoms in the overlayer is altered in this direction while remaining constant in the X direction. In this way, the corrugation pattern of (110) is preserved. For this reason, to account for all possible overlayer patterns, exploration of the non-stoichiometric overlayers has been carried out in three different patterns: along the X-axis, along the Y-axis, and along both axes, with the latter referred to as the bidirectional pattern hereafter. The non-stoichiometric overlayer is defined by a parameter P, which is the ratio of the number of atoms per layer in the underlayer and the number of atoms per layer in the overlayer. We explored all three patterns for P values between 0.6 and 2. Due to cost requirements, the number of P values that can be explored with DFT is limited. Consequently, to study systems with P values near the stoichiometric ratio (P = 1), an IP was trained for the Au-Pt interface using NequIP, a software package developed by Batzner *et al.* for the training of equivariant graph neural network interatomic potentials.[3] Once trained, the IP is able to predict the structure and energy of a system involving thousands of atoms at reasonable computational cost. Inaccuracies in the IP may make the surface energies not directly comparable with the DFT-derived surface energies, as seen by the difference in some surface energies at P = 1 in Figures S11 and 5. However, the results between different systems, all calculated with IP, should be robust for elucidating relative trends.

For the Y-axis search for a solution, DFT determined that, independently of the number of Pt MLs, the most stable overlayer is the stoichiometric solution (P = 1), see Figure S11. The same

conclusion was obtained for the X-axis and bidirectional searches, see Figure S11. In the bidirectional case, there is an exception when considering 1 ML of Pt, whereby the most stable structure was characterized by P = 1.78, however, the resulting surface is not a full ML of Pt, but rather a (110) surface with a mixture of Pt and Au atoms (inset of Figure S11c). Importantly, the surface energy of that solution is very close to the one for the P = 1 solution. Accordingly, we consider that the most stable overlayer for 1ML remains the stoichiometric solution. Interestingly, the stoichiometric solution does not imply a symmetric arrangement of the Pt overlayer on top of the Au(110) surface. As illustrated in Figure S10b for 1 ML of Pt, the top Pt overlayer shifts to occupy the interstitial sites on the Au(110) surface. This phenomenon was also observed to a varying degree for all stoichiometric solutions with 1−4 Pt MLs (Figure S10c).

Similarly, the most stable solution determined with IP for the 1 ML of Pt case was the stoichiometric solution and its structure was in agreement with the DFT-determined one. On the other hand, our IP analysis determined that with increasing number of Pt MLs (from 2 to 4), there is a shift of the most stable overlayer pattern from the stoichiometric solution (P = 1) to an overlayer with a P value equal to 0.94, indicating the existence of a moiré pattern (Figure S10d). The possibility of moiré patterns is reinforced by the structural agreement found between the IP and DFT for the stoichiometric solutions, where both predict the shifted Pt overlayer (Figure S10, b and c) as the most stable structure. Furthermore, because of computational cost, it is impossible to explore structures with P = 0.94 with DFT, an equivalent definition of ejection energy at the IP level of theory was used.

Next, the ejection energy was calculated for the most stable overlayer pattern with DFT and IP. Figure 5 shows that, per DFT, the ejection of a Pt atom from a stoichiometric Au@Pt surface is more difficult than that from a Pt(110) slab model surface, and at least 2.65 eV more difficult than the ejection of an Au atom from a Au(110) slab model surface, which is in agreement with our experimental observation. Moreover, as the number of Pt MLs on the Au surface increases, the Pt atom ejection energy keeps increasing and stabilizes when the number of Pt ML becomes 3 or 4. Importantly, at room temperature, every 0.06 eV in energy translates to an order of magnitude in dissolution rates. Notably, a similar trend for Au@Pt overlayers was observed in the (111) geometry.[4] Specifically, Lopes *et al.* observed that for non-stoichiometric overlayers of Pt on Au(111), the ejection energy of the Pt atom for cases with 1 and 4 MLs of Pt was higher than for ejection from monometallic Pt(111). We attribute this increase to the bonding between Au and Pt

at the interface in the Au@Pt system and the stabilization imparted by the strain fields induced by overlaying Pt on Au.

Since the DFT analysis could accurately describe the P = 1 solutions, we focused our IP-based analysis on the most stable overlayer systems centered at P = 0.94 for the cases with 2, 3, and 4 MLs of Pt overlayers. The IP-derived ejection energies are summarized in Figure S12a. In all cases, we find that the Au@Pt systems all have Pt-atom ejection energies significantly higher than Au-atom ejection energy from Au(110), which is calculated to be ~3.46 eV. In the case of 2 ML of Pt on Au(110), it requires 5.4 eV for ejecting a Pt atom and that value increases to 6.83 and 6.89 eV for 3 and 4 MLs of Pt, respectively. This result is in agreement with our experimental observations, pointing to significant higher stability of the Pt-coated AuRD.

Density Functional Theory Calculations. All systems have been studied with periodic density functional theory (DFT) calculations employing the Perdew-Burke-Ernzerhof (PBE) exchange-correlation functional within the generalized gradient approximation (GGA).[5] All DFT calculations were performed using the *Vienna Ab initio simulation package* (VASP).[6] The Kohn-Sham equations were solved within a plane-wave basis set with a cutoff of 230 eV. The effect of core electrons on the valence region was included through the projected augmented wave method as implemented by Kresse and Joubert.[7] Monometallic surfaces and bulk systems for Au and Pt were modelled using 3x3 supercells and one unit cell (4 atoms), respectively. For all monometallic surface models, a total of 6 atomic layers were included, where the two bottommost layers were frozen at their bulk-truncated positions during energy optimization and the four topmost layers were fully relaxed. For systems with Au/Pt interfaces, the supercell size varies from 3x3 to up to 6x6, where the actual size depends on the P value and the explored pattern (X, Y, and bidirectional). Regardless, all interface models include 4 Au atomic layers where the two bottommost Au layers were frozen during the energy optimization and the two topmost layers were fully relaxed. The Pt overlayers were always allowed to relax. The numerical integration was carried out in the reciprocal space using automatically generated Monkhorst-Pack grids by Python Materials Genomics (pymatgen) with a minimum density of 4500 k-points / number of atoms in the supercell for all surface models.[8,9] For bulk systems, the reciprocal space was sampled using a 15x15x15 k-points grid. All surface slab models include 15 Å of vacuum between successive slabs in the Z direction. The optimized lattice constant for bulk Au and Pt is 4.174 Å and 3.978 Å, respectively. These compare well with experimental lattice constants of 4.079 Å and 3.923 Å,

respectively.[10]

The possibility for non-stoichiometric overlayer structures along the X-direction, Y-direction, and simultaneously along both X and Y directions were explored. To limit the parameter space, no angle-dependent solutions were explored for this study, *i.e.*, the angular relative orientation of the support (Au) and overlayer (Pt) was preserved for all models. To keep the computational cost manageable, only up to 6x6 supercells were considered for the Au support. These limitations restrict the number of P values that can be evaluated. For example, only 7 solutions between 0.6 > P > 2 can be found for a bidirectional overlayer pattern, where most of the solutions are concentrated at the extremes of the interval, leading to a significant part of the interval near P = 1 remaining unexplored. This inconvenience is usually circumvented using combinations of root unit cells $\sqrt{x} \times \sqrt{x}$, where x is the number of atoms in the surface, however, no solutions are found for P values within the proposed range that do not imply a rotational angle between the Au underlayer and Pt overlayer. Table S1 summarizes the surface energies calculated for all P values evaluated for each of the patterns at the DFT level.

The surface energy (Γ) was defined as:

$$\Gamma = \frac{E_{Tot} - N_{Pt} \times E_{Pt_{bulk}} - N_{Au} \times E_{Au_{bulk}}}{2 \times A}$$

where E_{Tot} is the total energy of the interface-containing slab (Au@Pt), N_{Pt} is the number of Pt atoms in the slab, N_{Au} is the number of Au atoms in the slab, $E_{Pt_{bulk}}$ is the energy of a Pt atom in the bulk, $E_{Au_{bulk}}$ is the energy of a Au atom in the bulk, and A is the area of each exposed surface in the slab model (each slab contains two exposed surfaces).

The ejection energy was calculated as follows:

$$E_{ejec} = E_x + E_{Defect} - E_{Tot}$$

where E_x (x = Au or Pt) is the gas-phase energy of an Au or Pt atom, E_{Defect} is the energy of the slab of interest where one Au or Pt has been ejected from the surface, and E_{Tot} is the energy of the slab with no defect in it.

Machine Learning Interatomic Potential (IP). An equivariant graph neural network was trained using the NequIP python package (version 0.5.6).[3] The dataset used to train the model consisted of 3281 configurations evaluated with DFT calculations. This dataset consisted of 585 configurations of monometallic Pt and Au bulk structures and structures presenting (111) facets,

43 configurations from an *ab initio* molecular dynamics run of a Pt on Au(110) system, and 2653 configurations from geometry optimization calculations from stoichiometric and non-stoichiometric Pt on Au(110) overlayer structures. This dataset was randomly split into training, validation, and test sets consisting of 1000, 200, and 2081 configurations, respectively.

The trained model consisted of three interaction blocks, one radial layer, and a cutoff radius of 6.0 Å. The model was trained for 1500 epochs. The mean absolute error per atom for the training, validation, and test sets for the trained model were 0.39 meV/atom, 0.57 meV/atom, and 0.50 meV/atom, respectively. Geometry optimizations were performed using the Atomic Simulation Environment python package with the LBFGS method until all forces were below 0.03 eV/Å.[11,12]

The surface energy was computed in the same way as in the DFT calculations. The ejection energy requires a different approach than what was used in the DFT calculations, since the IP is incapable of calculating the energy of a single atom in the gas phase. The ejection energy at the IP level of theory was defined as:

$$E_{ejec\_IP} = (E_{x_{bulk}} - E_{coh(x)_{DFT}}) + E_{Defect} - E_{Tot}$$

where $E_{x_{bulk}}$ (x = Au or Pt) is the energy of a Au or Pt atom in the bulk, $E_{coh(x)_{DFT}}$ is the cohesive energy of the Au or Pt calculated at the DFT level, E_{Defect} is the energy of the surface of interest where one Au or Pt has been ejected, and E_{Tot} is the energy of the defect-free slab. $E_{x_{bulk}} - E_{coh(x)_{DFT}}$ approximates the gas-phase energy of a single atom at the IP level of theory. The DFT-calculated cohesive energy was defined as:

$$E_{coh(x)_{DFT}} = E_{x_{bulk(DFT)}} - E_{x_{gas(DFT)}}$$

where $E_{x_{bulk(DFT)}}$ (x = Au or Pt) is the energy of a Au or Pt atom in the bulk and $E_{x_{gas(DFT)}}$ is the energy of a Au or Pt atom in the gas-phase, both calculated with DFT.

Ejection Energies. For both IP and DFT calculations, the ejection energy for all non-equivalent atoms in the surface of the models was considered. For highly symmetric systems, such as the stoichiometric solutions for Au@Pt overlayers, the number of inequivalent atoms is limited. Nevertheless, non-stoichiometric systems are characterized by much lower symmetry, which in turn leads to a rapid increase in the number of inequivalent surface atoms. This is the case for the non-stoichiometric Au@Pt systems (P = 0.94), which are predicted to be more stable than the

stoichiometric solution through the IP (for 2, 3, and 4 MLs of Pt). The easiest atom to be ejected corresponds to an ejection energy that is reported in Figure S10d. The ejection energy for all other possible surface atom ejections is summarized in three histograms (Figure S12b), one for each number of MLs of Pt on top of Au.

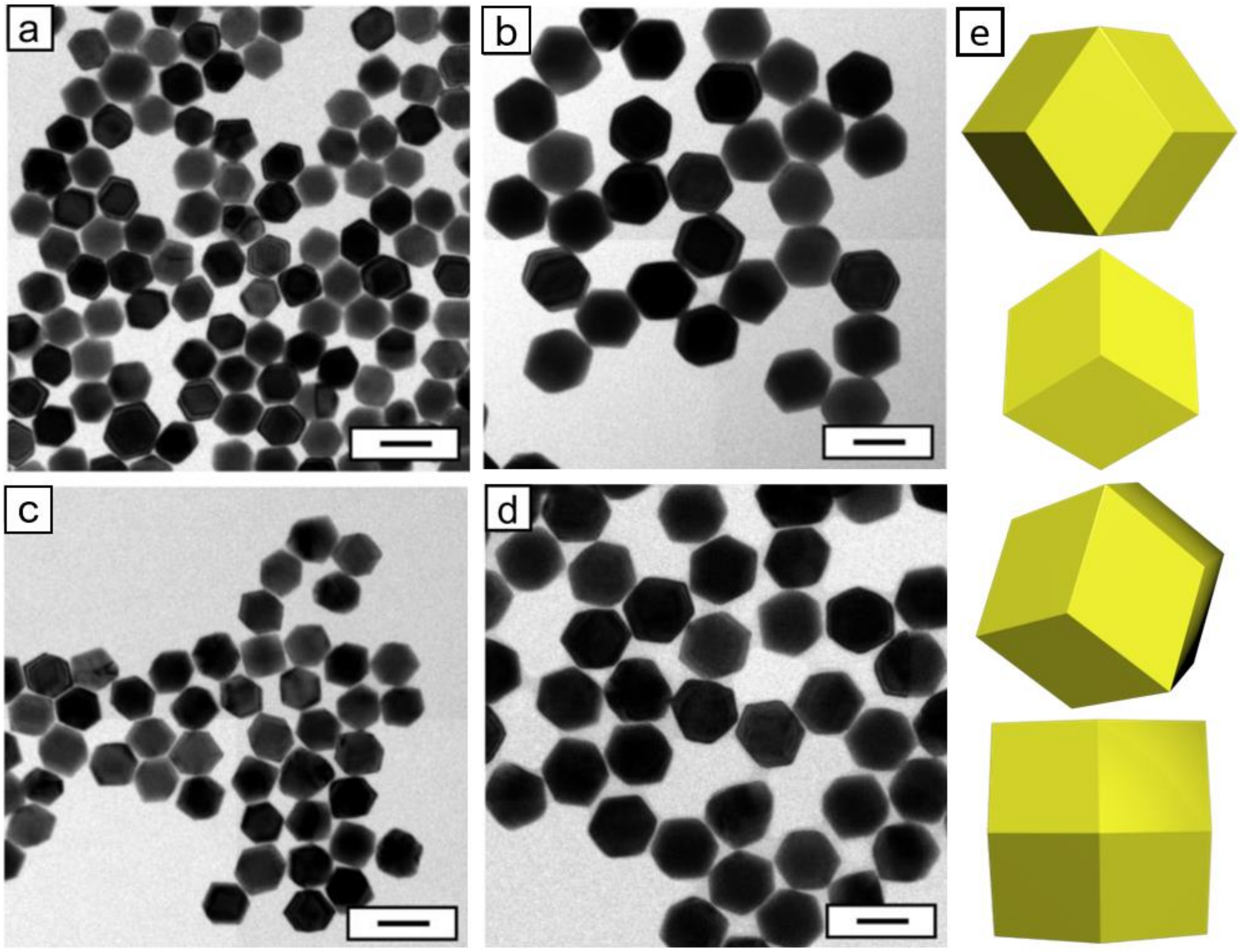

Figure S1. (a, c) TEM images of the 18-nm AuRD (a) before and (c) after coating with Pt. (b, d) TEM images of the 32-nm AuRD (b) before and (d) after coating with Pt. (e) Models of RD at different orientations. Scale bars: 50 nm.

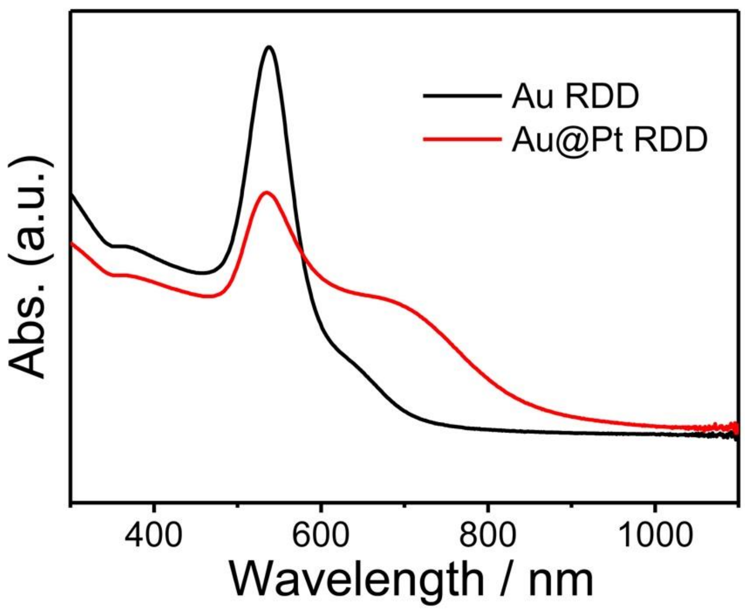

Figure S2. UV-vis spectra recorded from ethanol suspensions of the 18-nm AuRD before and after coating with Pt.

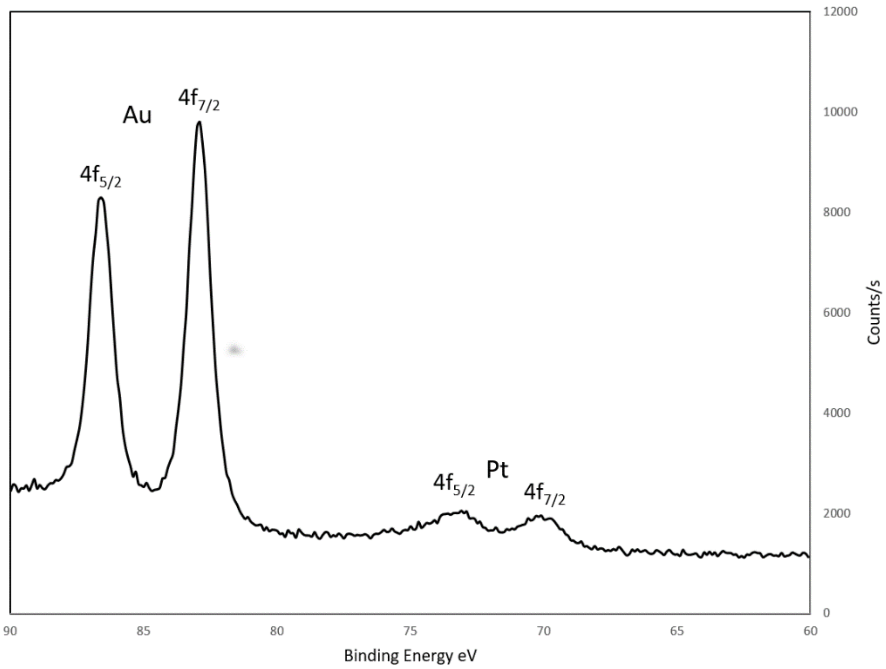

Figure S3. Au and Pt 4f XPS spectra recorded from the 18-nm AuRD coated with Pt. All the peaks are assigned and labeled accordingly.

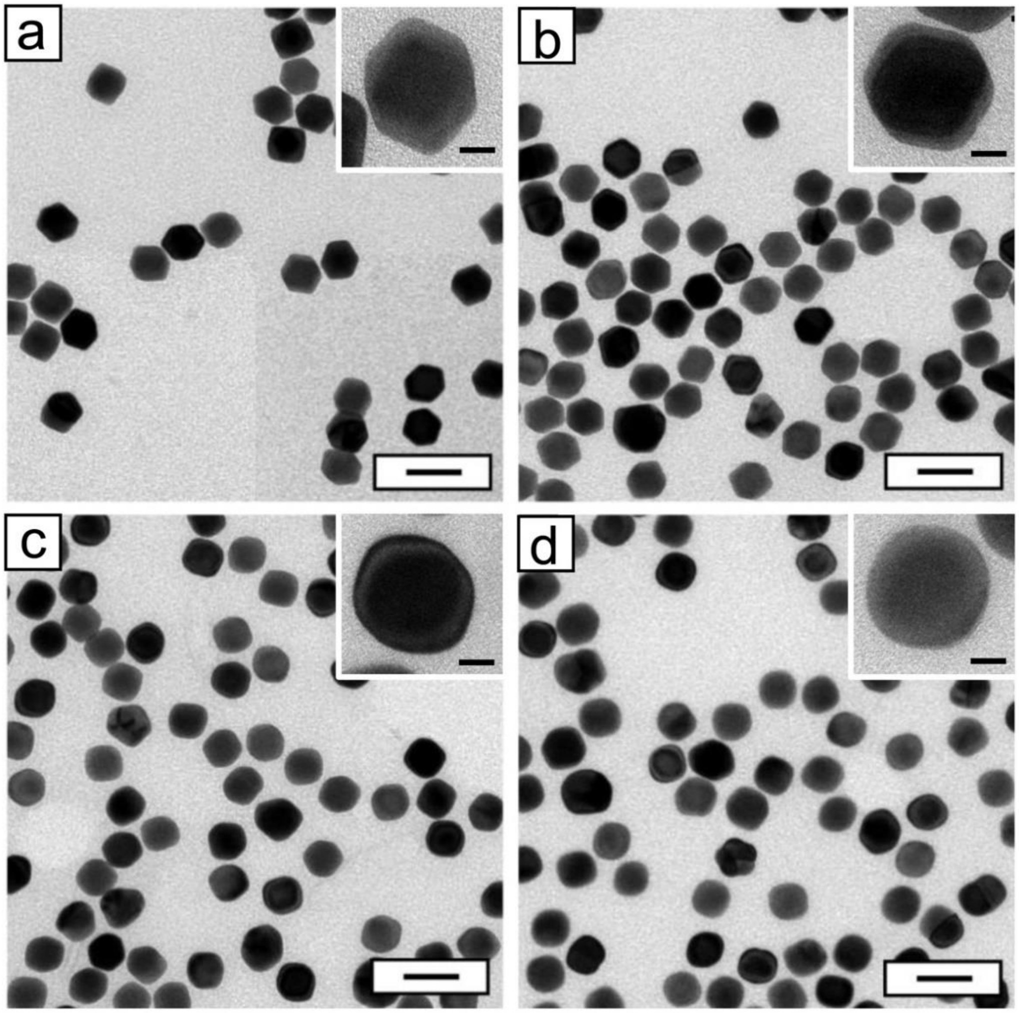

Figure S4. TEM images of the 18-nm AuRD after being heated in DMF at (a) 60, (b) 80, (c) 100, and (d) 120 ℃, respectively, and for 3 h at each temperature. Scale bars: 50 nm. Scale bars in the insets: 10 nm.

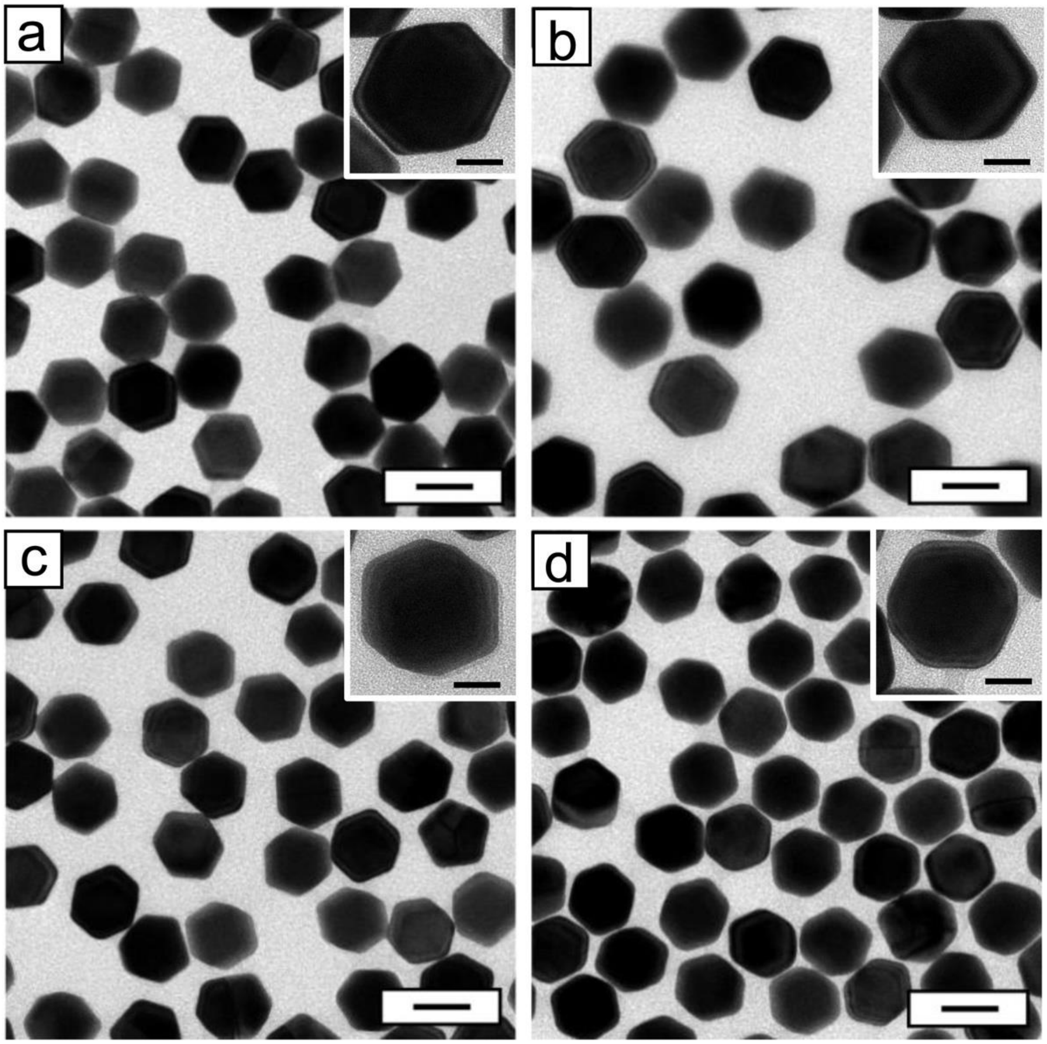

Figure S5. TEM images of the 32-nm AuRD after being heated in DMF at (a) 60, (b) 80, (c) 100, and (d) 120 ℃, respectively, and for 3 h at each temperature. Scale bars: 50 nm. Scale bars in the insets: 20 nm.

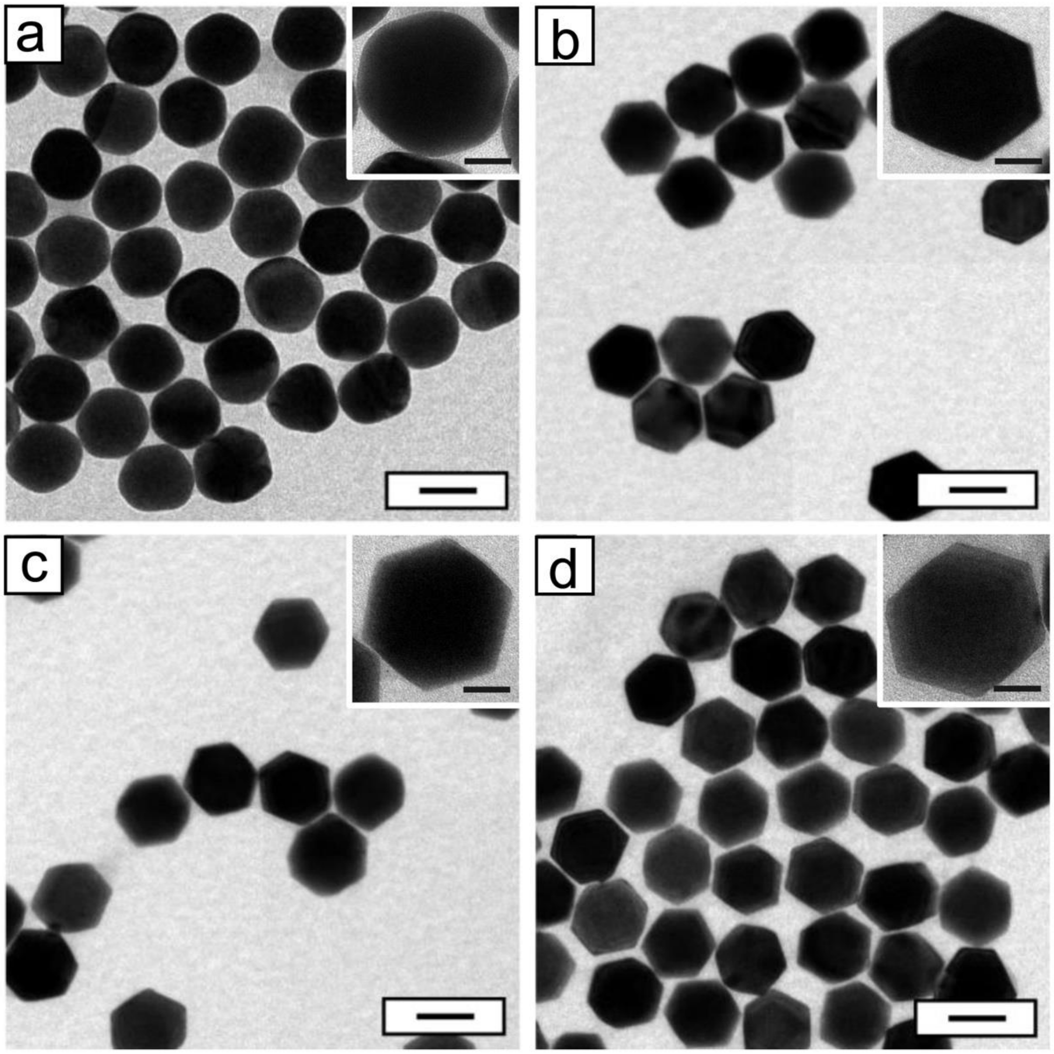

Figure S6. (a) TEM image of the 32-nm AuRD after being heated in DMF at 130 ℃ for 3 h. (b-d) TEM images of the Pt-coated 32-nm AuRD after being heated in DMF at (b) 130, (c) 150, (d) 180 ℃, respectively, and for 3 h at each temperature. Scale bars: 50 nm. Scale bars in the insets: 10 nm.

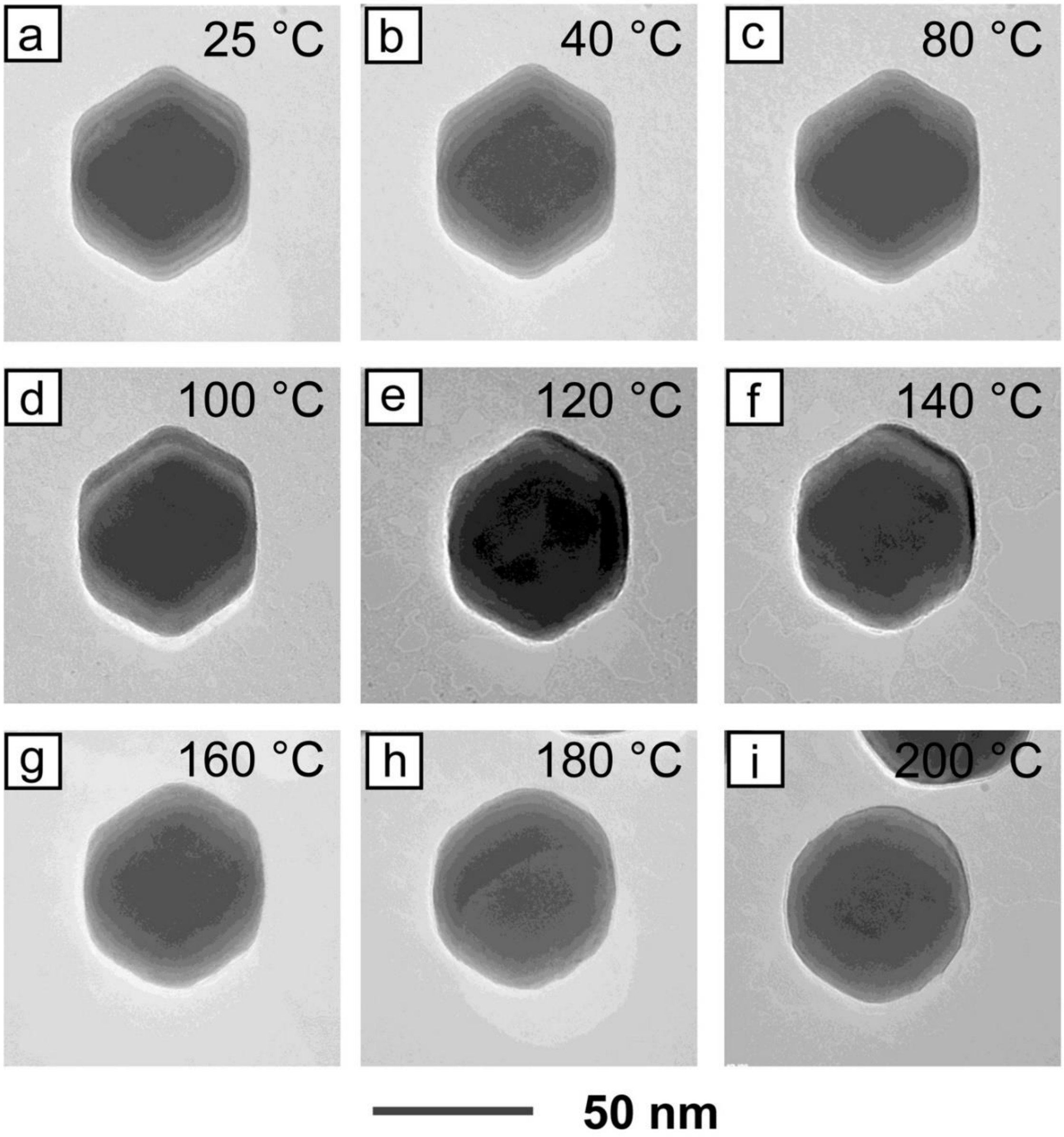

Figure S7. *In situ* TEM images recorded from the 32-nm AuRD upon heating consecutively at different temperatures for 20 min: (a) 25, (b) 40, (c) 60, (d) 80, (e) 100, (f) 120, (g) 160, (h) 180, and (i) 200 °C. The original shape was lost at 140 °C and the nanocrystal evolved into a nanosphere at the end.

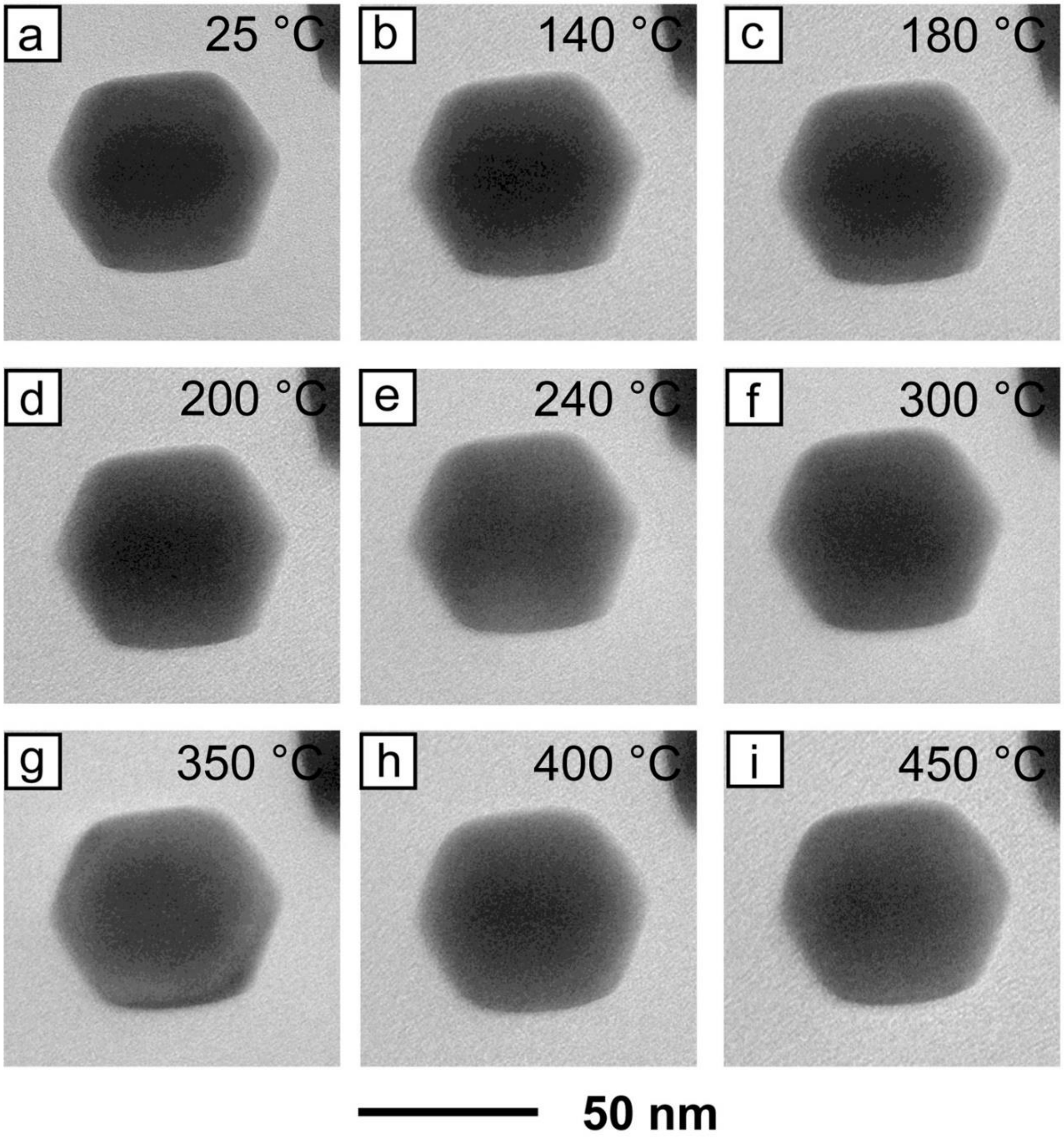

Figure S8. *In situ* TEM images recorded from the 32-nm Pt-coated AuRD upon heating consecutively at different temperatures for 20 min: (a) 25, (b) 140, (c) 180, (d) 200, (e) 240, (f) 300, (g) 350, (h) 400, and (i) 450 °C. It is worth noting that the original shape was largely retained up to 400 °C.

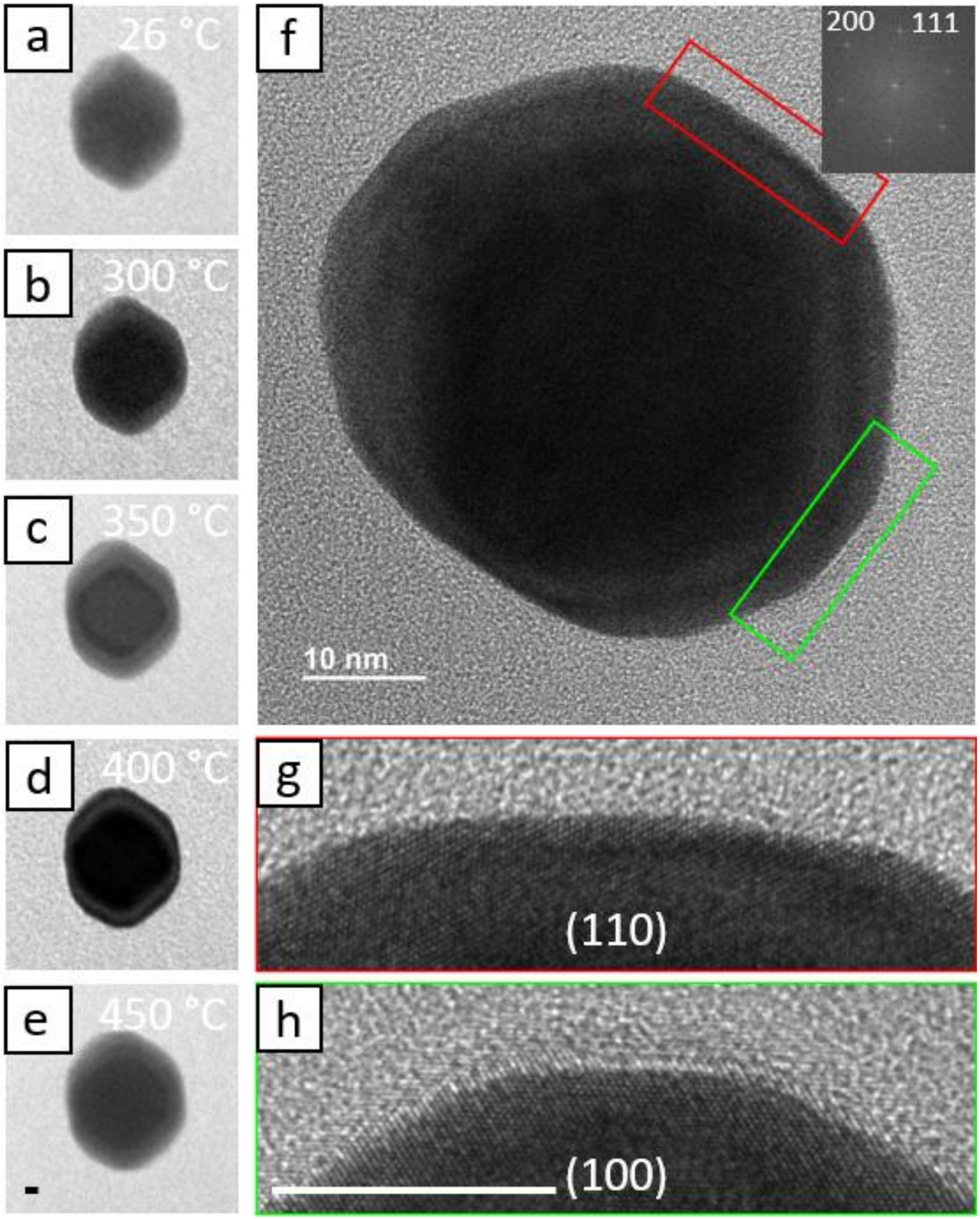

Figure S9. (a-e) *In situ* TEM images recorded from the 32-nm Pt-coated AuRD upon heating consecutively at different temperatures for 10 min: (a) 26, (b) 300, (c) 350, (d) 400, and (e) 450 °C. (f) HRTEM image taken along the [011] zone axis of the same 32-nm Pt-coated AuRD after heating at 450 °C with an inset of the corresponding labeled FFT pattern. (g, f) HRTEM images of the regions indicated by (g) red and (h) green outlines in (f). Scale bars: 10nm.

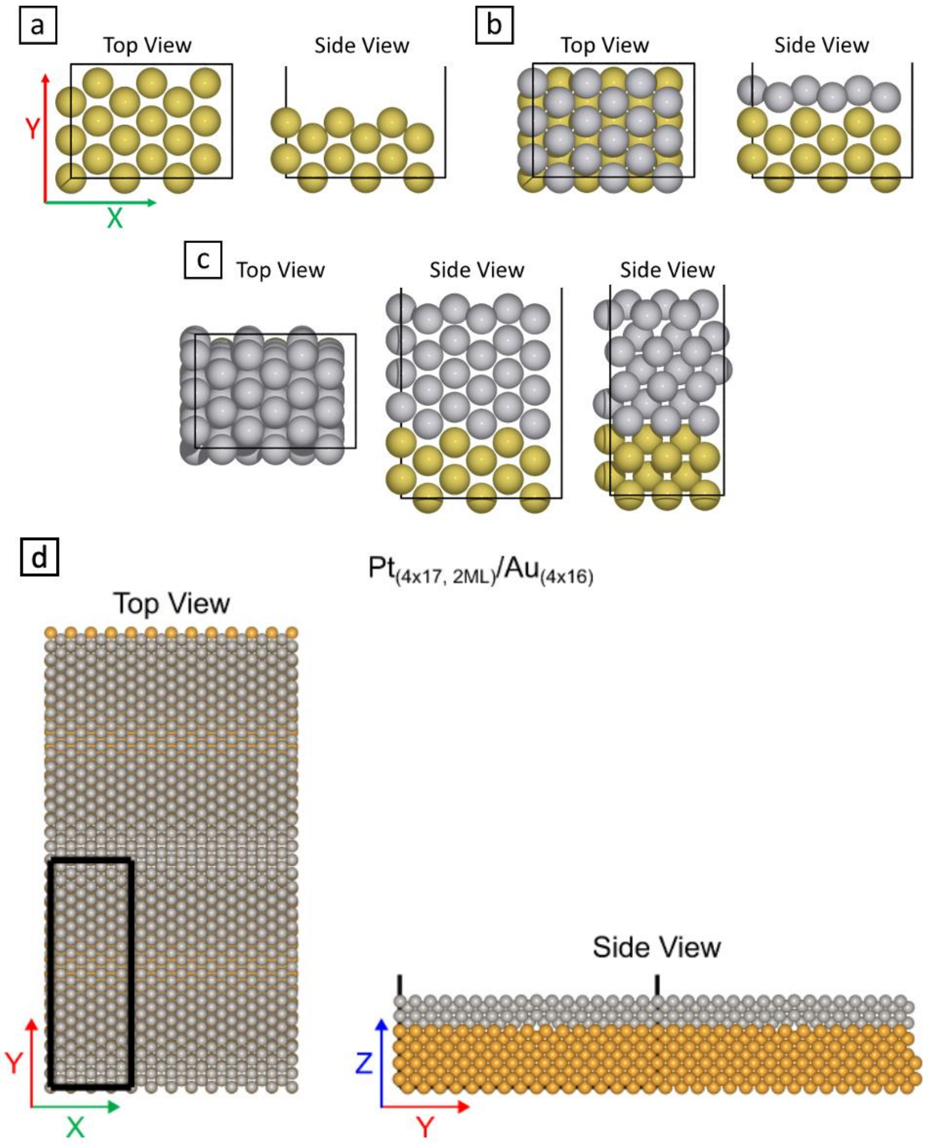

Figure S10. Illustrations depicting: (a) the structure of a monometallic Au(110) surface, (b) a stoichiometric Pt ML supported on Au(110), and (c) 4 stoichiometric MLs of Pt supported on Au(110) as optimized with DFT. Red and green arrows in (a) indicate the direction along which non-stoichiometric overlayers were studied. (d) Top view and side view of the non-stoichiometric overlayer structure for 2 ML of $Pt_{(4x17)}$ supported on $Au_{(4x16)}$. The dark lines indicate the outline of the unit cell. Three repetitions of the unit cell in the X-direction and two in the Y-direction are shown to visualize the long-range patterns of the structure. Yellow and grey spheres correspond to Au and Pt atoms, respectively.

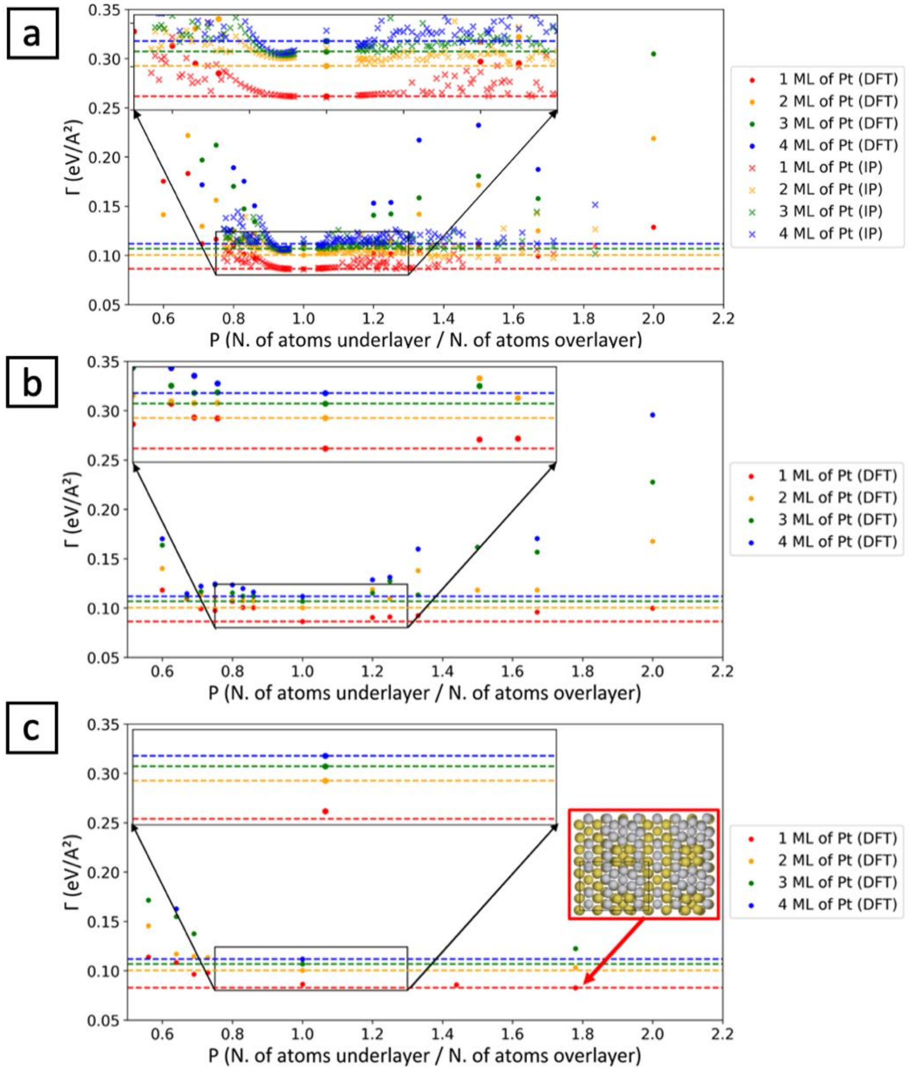

Figure S11. Calculated surface energies (Γ) for Pt overlayers along the Y axis (a), X axis (b), and bidirectional (c) patterns as a function of the P-value (the ratio of the number of atoms per layer in the underlayer and the number of atoms in the overlayer). Circles and crosses refer to Γ calculated with DFT and IP, respectively. Red, yellow, green, and blue colors indicate the results for 1, 2, 3, and 4 MLs of Pt on a Au(110) surface. The dashed horizontal lines indicate the lowest Γ (per DFT), which in all but one case corresponds to the stoichiometric solution (P = 1). The inset in (c) illustrates the structure of the most stable Pt overlayer when only 1 ML of Pt is supported on Au(110). Yellow and grey spheres of the inset in (c) correspond to Au and Pt atoms, respectively. In each panel, the small parallelogram near its bottom is blown-up closer to the top of it, for between resolution.

Table S1. Summary of surface energies (Γ) calculated with DFT for all studied patterns (Y, X, and bidirectional) with 1 ML of Pt. For each pattern, the left-hand column indicates the studied P value (in bold) and the supercell combination (Au x Au)/(Pt x Pt) used to obtain the corresponding P value. For each pattern, the right column indicates the surface energy (eV/Å^2) of 1 ML of Pt on Au(110) for the corresponding P value in the left column. Results are presented from top to bottom of the table with increasing value of P.

**Y pattern**		**X pattern**		**Bidirectional pattern**	
**P value** (Au x Au)/(Pt x Pt)	**Γ (eV/Å^2)** 1 ML of Pt	**P value** (Au x Au)/(Pt x Pt)	**Γ (eV/Å^2)** 1 ML of Pt	**P value** (Au x Au)/(Pt x Pt)	**Γ (eV/Å^2)** 1 ML of Pt
**0.60** (3x3)/(3x5)	0.175	**0.60** (3x3)/(5x3)	0.118	**0.56** (3x3)/(4x4)	0.114
**0.67** (3x4)/(3x6)	0.183	**0.67** (4x3)/(6x3)	0.110	**0.64** (4x4)/(5x5)	0.108
**0.71** (3x5)/(3x7)	0.112	**0.71** (5x3)/(7x3)	0.099	**0.69** (5x5)/(6x6)	0.097
**0.75** (3x6)/(3x8)	0.116	**0.75** (6x3)/(8x3)	0.097	**0.73** (6x6)/(7x7)	0.098
**0.80** (3x4)/(3x5)	0.110	**0.80** (4x3)/(5x3)	0.107	**1.00** (3x3)/(3x3)	0.086
**0.83** (3x5)/(3x6)	0.101	**0.83** (5x3)/(6x3)	0.101	**1.44** (6x6)/(5x5)	0.086
**0.86** (3x6)/(3x7)	0.097	**0.86** (6x3)/(7x3)	0.100	**1.78** (4x4)/(3x3)	0.083
**1.00** (3x3)/(3x3)	0.086	**1.00** (3x3)/(3x3)	0.086		
**1.20** (3x6)/(3x5)	0.102	**1.20** (6x3)/(5x3)	0.090		
**1.25** (3x5)/(3x4)	0.102	**1.25** (5x3)/(4x3)	0.091		
**1.33** (3x4)/(3x3)	0.105	**1.33** (4x3)/(3x3)	0.092		
**1.50** (3x6)/(3x4)	0.111	**1.50** (6x3)/(4x3)	-		
**1.67** (3x5)/(3x3)	0.099	**1.67** (5x3)/(3x3)	0.096		
**2.00** (3x4)/(3x2)	0.129	**2.00** (4x3)/(2x3)	0.100		

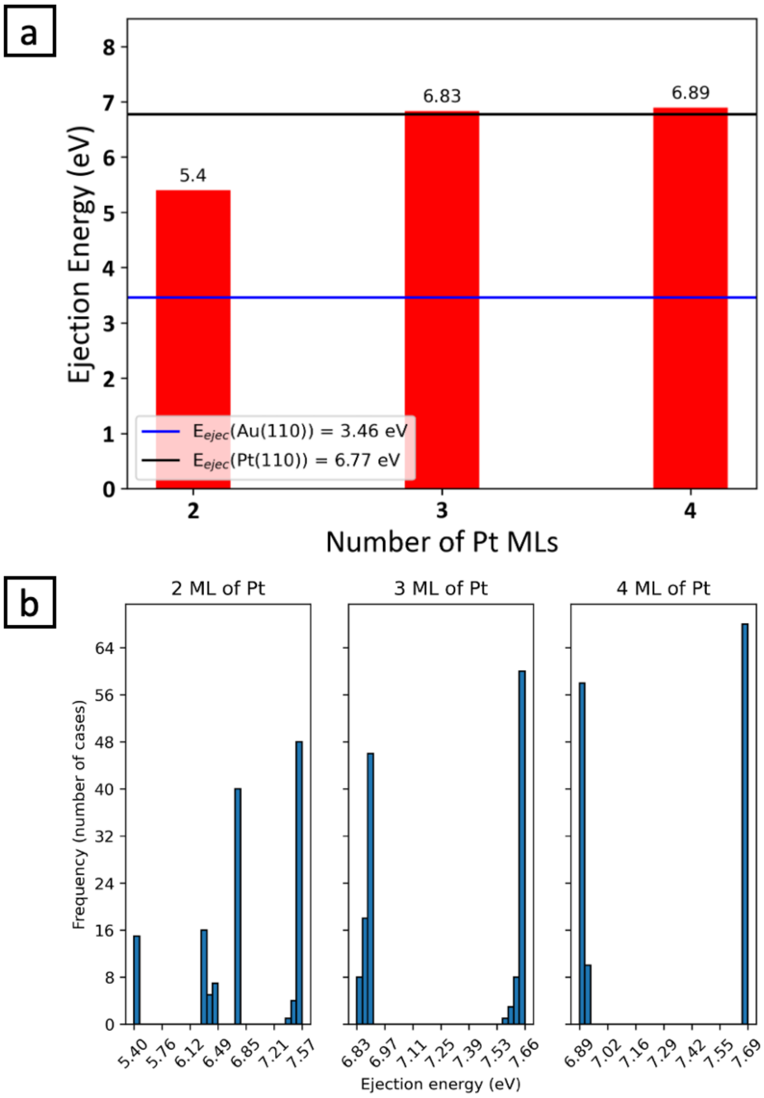

Figure S12. (a) IP-calculated ejection energies for Pt atoms in non-stoichiometric Pt overlayers (P = 0.94) supported on Au(110). For comparison, blue and black horizontal lines indicate the IP-calculated ejection energy for Au and Pt atoms from monometallic Au(110) and Pt(110) surfaces, respectively. Values correspond to the easiest ejection of a surface atom for each system. (b) Histogram of IP-calculated ejection energies for the three surfaces described in (a). The first entry in each panel is shown in (a).

References

1. Zheng, Y.; Ma, Y.; Zeng, J.; Zhong, X.; Jin, M.; Li, Z.-Y.; Xia, Y., Seed-Mediated Synthesis of Single-Crystal Gold Nanospheres with Controlled Diameters in the Range 5–30 nm and their Self-Assembly upon Dilution. *Chem. – Asian J.* **2013,** *8*, 792-799.
2. Zhao, X.; Pawlik, V. D.; Huo, D.; Zhou, S.; Yang, B.; Xia, Y., Mechanistic Study of Seed-Mediated Growth of Gold Rhombic Dodecahedra. *J. Phys. Chem. C* **2021,** *125*, 27394-27402.
3. Batzner, S.; Musaelian, A.; Sun, L.; Geiger, M.; Mailoa, J. P.; Kornbluth, M.; Molinari, N.; Smidt, T. E.; Kozinsky, B., E(3)-Equivariant Graph Neural Networks for Data-Efficient and Accurate Interatomic Potentials. *Nat. Commun.* **2022**, *13*, 1–11.
4. Lopes, P. P.; Li, D.; Lv, H.; Wang, C.; Tripkovic, D.; Zhu, Y.; Schimmenti, R.; Daimon, H.; Kang, Y.; Snyder, J.; Becknell, N.; More, K. L.; Strmcnik, D.; Markovic, N. M.; Mavrikakis, M.; Stamenkovic, V. R., Eliminating dissolution of platinum-based electrocatalysts at the atomic scale. *Nat. Mater.* **2020,** *19*, 1207-1214.
5. Perdew, J. P.; Burke, K.; Ernzerhof, M., Generalized Gradient Approximation Made Simple. *Phys. Rev. Lett.* **1996**, *77*, 3865.
6. Kresse, G.; Furthmüller, J., Efficient iterative schemes for *ab initio* total-energy calculations using plane-waave basis set. *Phys. Rev. B* **1996**, *54*, 11169.
7. Kresse, G.; Joubert, D., From ultrasoft pseudopotentials to the projector augmented-wave method. *Phys. Rev. B* **1999**, *59*, 1758-1775.
8. Monkhorst, H. J.; Pack, J. D., Special points for Brillouin-zone integrations. *Phys. Rev. B* **1976**, *13*, 5188.
9. Ong, S. P.; Richards, W. D.; Jain, A.; Hautier,G.; Kocher, M.; Cholia, S.; Gunter, D.; Chevrier, V. L.; Persson, K. A.; Ceder, G., Python materials genomics (pymatgen): a robust, open-source python library for materials analysis. *Comput. Mater. Sci.* **2013**, *68*, 314-319.
10. Haynes, W. M. *CRC Handbook of Chemistry and Physics, 95th ed.; CRC Press: New York, 2014–2015.*
11. Larsen, A. H.; Mortensen, J. J.; Blomqvist, J.; Castelli, I. E.; Christensen, R.; Dułak, M.; Friis, J.; Groves, M. N.; Hammer, B.; Hargus, C.; Hermes, E. D.; Jennings, P. C.; Bjerre Jensen, P.; Kermode, J.; Kitchin, J. R.; Kolsbjerg, E. L.; Kubal, J.; Kaasbjerg, K.;

Lysgaard, S.; Maronsson, J. B.; Maxson, T.; Olsen, T.; Pastewka, L.; Peterson, A.; Rostgaard, C.; Schiøtz, J.; Schütt, O.; Strange, M.; Thygesen, K. S.; Vegge, T.; Vilhelmsen, L.; Walter, M.; Zeng, Z.; Jacobsen, K. W., The atomic simulation environment—a Python library for working with atoms. *J. Phys.: Condens. Matter* **2017**, *29*, 273002.

12. Liu, D. C.; Nocedal, J., On the limited memory BFGS method for large scale optimization. *Math Program* **1989**, *45*, 503–528.
